# C9orf72/ALFA-1 controls TFEB/HLH-30-dependent metabolism through dynamic regulation of Rag GTPases

**DOI:** 10.1371/journal.pgen.1008738

**Published:** 2020-04-13

**Authors:** Yon Ju Ji, Janet Ugolino, Tao Zhang, Jiayin Lu, Dohoon Kim, Jiou Wang

**Affiliations:** 1 Department of Biochemistry and Molecular Biology, Bloomberg School of Public Health, Johns Hopkins University, Baltimore, MD, United States of America; 2 Department of Neuroscience, School of Medicine, Johns Hopkins University, Baltimore, MD, United States of America; University of California, San Francisco, UNITED STATES

## Abstract

Nutrient utilization and energy metabolism are critical for the maintenance of cellular homeostasis. A mutation in the *C9orf72* gene has been linked to the most common forms of neurodegenerative diseases that include amyotrophic lateral sclerosis (ALS) and frontotemporal dementia (FTD). Here we have identified an evolutionarily conserved function of C9orf72 in the regulation of the transcription factor EB (TFEB), a master regulator of autophagic and lysosomal genes that is negatively modulated by mTORC1. Loss of the *C*. *elegans* orthologue of C9orf72, ALFA-1, causes the nuclear translocation of HLH-30/TFEB, leading to activation of lipolysis and premature lethality during starvation-induced developmental arrest in *C*. *elegans*. A similar conserved pathway exists in human cells, in which C9orf72 regulates mTOR and TFEB signaling. C9orf72 interacts with and dynamically regulates the level of Rag GTPases, which are responsible for the recruitment of mTOR and TFEB on the lysosome upon amino acid signals. These results have revealed previously unknown functions of C9orf72 in nutrient sensing and metabolic pathways and suggest that dysregulation of C9orf72 functions could compromise cellular fitness under conditions of nutrient stress.

## Author summary

An expansion of repeated nucleotides in the non-coding region of the *C9orf72* gene has been linked to the neurodegenerative diseases amyotrophic lateral sclerosis (ALS) and frontotemporal dementia (FTD). The repeat expansion leads to a reduced expression of the *C9orf72* gene and loss of function of the C9orf72 protein may contribute to the pathogenesis. In this study, we identified a new mechanism through which C9orf72 influences nutrient sensing, autophagy, and metabolism. In the multi-cellular organism *Caenorhabditis elegans*, the C9orf72 orthologue regulates the activity of TFEB, a crucial transcriptional regulator of autophagic and lysosomal genes, through which the lipid metabolism and survival are influenced especially under nutrient stress conditions. The regulatory effect of C9orf72 on TFEB is conserved in mammals, and this is mediated by the dynamic regulation of the Rag GTPases by C9orf72. Given the critical role of the Rag GTPases in nutrient sensing and autophagy, we propose that the C9orf72 function is important for metabolic homeostasis in the cell and its deficiency can lead to compromised fitness under stress conditions.

## Introduction

Recognition of a hexanucleotide repeat expansion (HRE) in the *C9orf72* gene as the most common cause of the neurodegenerative diseases amyotrophic lateral sclerosis (ALS) and frontotemporal dementia (FTD) has opened avenues for understanding the molecular mechanisms of a number of neurological diseases [1, 2]. In addition to being the most common cause of ALS and FTD, two related neurodegenerative conditions [3], there is genetic evidence to suggest that the C9orf72 repeat expansion also contributes to Alzheimer’s disease [4–7], Huntington’s disease [8], and other neurological conditions, including multiple system atrophy [9], depressive pseudodementia [10], and bipolar disorder [11]. How the C9orf72 repeat expansion leads to neurodegeneration remains to be determined, although both gain-of-toxicity and loss-of-function mechanisms have been proposed. The gain-of-toxicity mechanisms involve both RNA and protein products generated from the expanded hexanucleotide repeats. However, the dysregulation of the C9orf72 protein as a result of the repeat expansion could contribute to the pathogenesis of the relevant diseases. Multiple studies have demonstrated that *C9orf72* RNA and protein levels are decreased in the brains and other tissues of ALS/FTD patients, who carry hundreds to thousands of the hexanucleotide repeats, when compared to normal individuals, who typically have only several repeats [12–16]. Moreover, loss of C9orf72 hypersensitizes cells to stress [17], and the haploinsufficiency of C9orf72 leads to neurodegeneration in human motor neurons [18].

Increasing evidence suggests that C9orf72 plays a role in the regulation of autophagic and lysosomal activity. Studies of the C9orf72 protein have revealed a DENN-like domain in its structure and its functions involved in membrane trafficking, the autophagy-lysosome pathway, autoimmunity, and metabolism [19–30]. C9orf72 has been shown to influence the initiation of autophagy via the serine/threonine-protein kinase ULK1 [23–26]. C9orf72 has also been reported to interact with several members of the Rab GTPase family, including Rab1, Rab5, Rab7, and Rab11, and may show activity characteristic of a guanine exchange factor [19, 28]. Moreover, C9orf72 has been shown to modulate the activity of mTORC1, a protein complex that regulates many cellular processes, including autophagy [22, 27]. We have previously reported that loss of C9orf72 protein causes the aberrant activation of transcription factor EB (TFEB), the master transcription factor for lysosomal genes by promoting its nuclear translocation [27]. CARM1, a histone arginine methyltransferase and a co-activator of TFEB, is recruited by C9orf72 to lysosomes for degradation under conditions of nutrient stress [30]. However, how C9orf72 regulates mTORC1 and TFEB signaling and downstream autophagic and lysosomal activity remains unknown.

Here we report the findings of a function of C9orf72 that is evolutionarily conserved from *C*. *elegans* to humans in the regulation TFEB signaling and downstream activity. While utilizing *C*. *elegans* as a model organism to study the conserved function of C9orf72, we identified a nutrient-dependent survival phenotype that is attributable to a defect in lipid metabolism. These phenotypes in *C*. *elegans* are dependent on the regulation of TFEB. In mammalian cells, C9orf72 was found to interact with Rag GTPases and regulate the activation of TFEB. These results suggest that the Rag GTPases are the key player in the mechanism through which C9orf72 regulates TFEB signaling and downstream metabolic processes.

## Results

### The *C*. *elegans* orthologue of C9orf72, *alfa-1*, is essential for survival in L1 diapause

**Fig 1:**
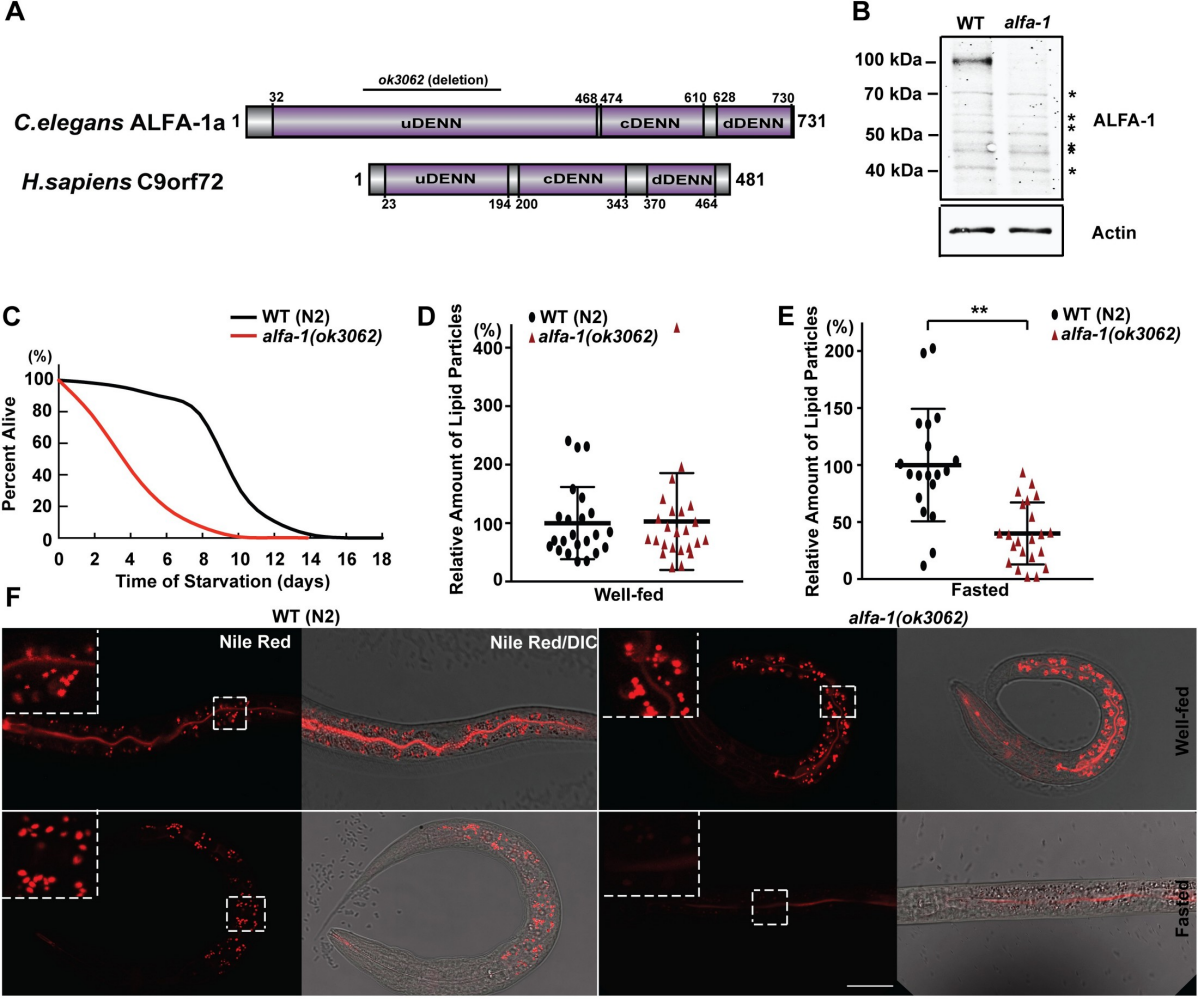
The *C*. *elegans* orthologue of C9orf72, *alfa-1*, regulates lipid metabolism at L1 diapause. (A) The domain structure of the *C*. *elegans* ortholog of C9orf72, *alfa-1*. The DENN domain structure was found using the Scanprosite tool in the ExPASy Bioinformatics Resource Portal with amino acid sequences of ALFA-1, isoform a. The deletion region of the *ok3062* allele is indicated by the bar. (B) Western blot analysis of ALFA-1. ALFA-1 runs at approximately 100 kDa in protein samples from wild-type animals. Asterisks indicate non-specific bands. (C) Percentage of L1 worms that survived to adulthood after starvation for the indicated time at L1. Survival of *alfa-1(ok3062)* after starvation was lower than that of wild-type *C*. *elegans*. (D) Quantification of the lipid particles stained with Nile Red in wild-type and *alfa-1(ok3062) C*. *elegans* under the well-fed condition. No significant difference was found in the number of Nile Red particles between N2 and *alfa-1(ok3062)*. [*p* = 0.8975, n = 23 for wild-type and n = 24 for *alfa-1(ok3062) C*. *elegans*]. (E) Quantification of the lipid particles stained with Nile Red in wild-type and *alfa-1(ok3062) C*. *elegans* under the starvation condition. The number of Nile Red particles in *alfa-1(ok3062)* was significantly lower than that of wild-type *C*. *elegans*. [**p<0.0001, n = 19 for wild-type and n = 22 for *alfa-1(ok3062) C*. *elegans*]. (F) Representative images of Nile Red staining of L1 worms. Upper panels show the Nile Red staining of wild-type and *alfa-1(ok3062) C*. *elegans* under well-fed conditions, and the lower panels show the staining patterns of the worms under starvation conditions. Enlarged images of the boxed areas are shown in each panel. Nile Red staining was decreased in *alfa-1(ok3062*) when compared to wild-type *C*. *elegans* under the starvation conditions. Distribution of data points is presented with mean ± SD. Scale bar: 20 μm.

The human C9orf72 has an orthologue in *C*. *elegans*, the F18A1.6 gene, also recently named as *alfa-1* (ALS/FTD-associated gene homolog 1) [31]. We analyzed the domain structure of ALFA-1 and found that it shares the same DENN domains with human C9orf72, including the uDENN, cDENN, and dDENN domains (Fig 1A). We obtained a mutant strain of the *alfa-1* gene with a mutant allele *(ok3062)* consisting of 486-bp deletion and 24-bp insertion in the region of exons 3 and 4. To study the nature of *alfa-1(ok3062)*, we generated a rabbit polyclonal antibody against ALFA-1. ALFA-1 was detected near the 100-kDa protein size standard in wild-type N2 *C*. *elegans* by western blot analysis (Fig 1B). As expected, no signal was detected in *alfa-1(ok3062)* near the 100-kDa size marker. Moreover, no additional smaller band was detected as being unique in *alfa-1(ok3062)*, suggesting that this mutant allele is a null allele. With this allele, we saw no gross defects at the adult stage, and the brood size was comparable to that in wild-type N2 (S1A Fig).

The L1 larva is the first developmental stage after *C*. *elegans* embryos are hatched; this stage lasts for several hours when nutrients are abundant. If the embryos are hatched in the absence of food, the worms can survive but are arrested at the L1 stage for a few weeks without the occurrence of any major morphological changes; this arrest is referred to as the L1 diapause. Consistent with a previous report [32], we found that *alfa-1(ok3062)* displayed a significantly decreased survival at the L1 stage under starvation conditions when compared to the N2 strain (Fig 1C). Since a key player in the insulin signaling pathway, *daf-16*, has been reported to regulate L1 diapause [33], we asked whether the regulation of L1 diapause by *alfa-1* is dependent on the insulin signaling pathway. To address this question, we performed a genetic interaction analysis between *alfa-1* and the key loss-of-function mutants in the insulin signaling pathway, including *daf-2(e1370)* and *daf-16(mu86)*, with reference to the survival of L1 worms under the starvation conditions (S1B Fig).

DAF-2 is an insulin-like receptor that inhibits the nuclear translocation of DAF-16, which is the FOXO transcription factor in *C*. *elegans* and controls the expression of genes regulating development and L1 diapause [33]. We first examined the genetic interaction between *alfa-1(ok3062)* and *daf-2(e1370)*. Unlike *alfa-1(ok3062)*, which showed decreased L1 survival under the starvation conditions when compared to N2, *daf-2(e1370)* alone showed increased survival. Interestingly, *alfa-1(ok3062);daf-2(e1370)* double mutants exhibited decreased survival similar to *alfa-1(ok3062)* alone (S1B Fig), indicating that *alfa-1* is epistatic to *daf-2*. Next, we generated and challenged the double mutant *daf-16(mu86);alfa-1(ok3062)* under the same starvation conditions. Unlike *alfa-1(ok3062)*, *daf-16(mu86)* did not show decreased L1 survival when they were starved for two days. However, the survival of *daf-16(mu86)* sharply decreased after starvation for four days, consistent with the previous reports [34]. Furthermore, *daf-16(mu86);alfa-1(ok3062)* exhibited decreased L1 survival when compared to *alfa-1(ok3062)* or *daf-16(mu86)* alone (S1C Fig), suggesting that *alfa-1* regulates L1 survival under starvation in parallel to *daf-16*. The Insulin-like signaling pathway in *C*. *elegans* is also well-known to regulate longevity, therefore we asked if *alfa-1(ok3062)* longevity is changed under normal conditions. Notably, unlike *daf-2(e1370)* and *daf-16(mu86)*, which regulated lifespan, *alfa-1(ok3062)* did not influence the lifespan of *C*. *elegans* under normal conditions (S1D Fig). In addition, the longevity of *alfa-1(ok3062);daf-2(e1370)* and *daf-16(mu86);alfa-1(ok3062)* double mutants was similar to that of *daf-2(e1370)* and *daf-16(mu86)* single mutants, respectively, suggesting that *alfa-1* does not regulate the longevity under normal condition. Taken together, *alfa-1* regulates specifically L1 survival under the nutrient stress.

### HLH-30/TFEB is abnormally activated upon loss of *alfa-1*/C9orf72

To understand the molecular changes as a result of the loss of *alfa-1*, we analyzed the transcriptome profiles of *alfa-1(ok3062)* mutants and WT controls by using microarrays. Because *alfa-1(ok3062)* showed decreased survival at L1 diapause under starvation, we decided to analyze the transcriptome at the L3/L4 stage after starvation. Both N2 and *alfa-1(ok3062) C*. *elegans* were synchronized at the larval stage. Then, the L3/L4 stage *C*. *elegans* under the well-fed conditions and another set of worms starved for 6 hr were harvested for RNA extraction and whole-transcriptome microarray analysis (S2A Fig). It was previously reported that 6-hr starvation was sufficient to induce transcriptomic changes in young adults [35]. The transcriptome profiling data were analyzed using the Ingenuity Pathway Analysis platform to identify the molecular function categories in which the differentially regulated genes were enriched. Under both well-fed and starvation conditions, the most significantly represented molecular function related to the changes as a result of the loss of *alfa-1* pointed to lipid metabolism (S2B–S2E Fig). Notably, amino acid metabolism is also highly relevant to the changes as a result of the loss of *alfa-1* in *C*. *elegans* under starvation conditions (S2D and S2E Fig). These data suggested that *alfa-1* may influence metabolism especially that of lipids in the intact worms.

Next, we asked whether the survival phenotype of the *alfa-1(ok3062)* mutants at L1 diapause is related to any changes in lipid metabolism. During L1 diapause, a metabolic change is needed to preserve the energy necessary for the long-term survival under starvation conditions. As a major energy source in the starvation conditions, the fat content is stored in the intestine of *C*. *elegans* and when in need digested in lysosomes. Therefore, the lysosome is an important organelle for regulating energy metabolism in the cell. Nile Red, a vital dye that has long been used to detect the lipid in the *C*. *elegans* intestine [36], was reported to stain the lipid in the lysosome-related organelles [37]. When L1 or adult worms in well-fed conditions were treated with Nile Red, we detected no differences between *alfa-1(ok3062)* and wild-type N2 control animals (Fig 1D–1F and S3A and S3B Fig). However, when L1 worms cultured under starvation conditions were stained with Nile Red, we observed a significantly lower signal in the *alfa-1(ok3062)* mutants than in the N2 controls (Fig 1E and 1F). These data suggest a defect in lipid metabolism in the lysosome-related organelles of *alfa-1(ok3062)* mutants that could explain the premature death of the L1 mutants under starvation conditions.

**Fig 2:**
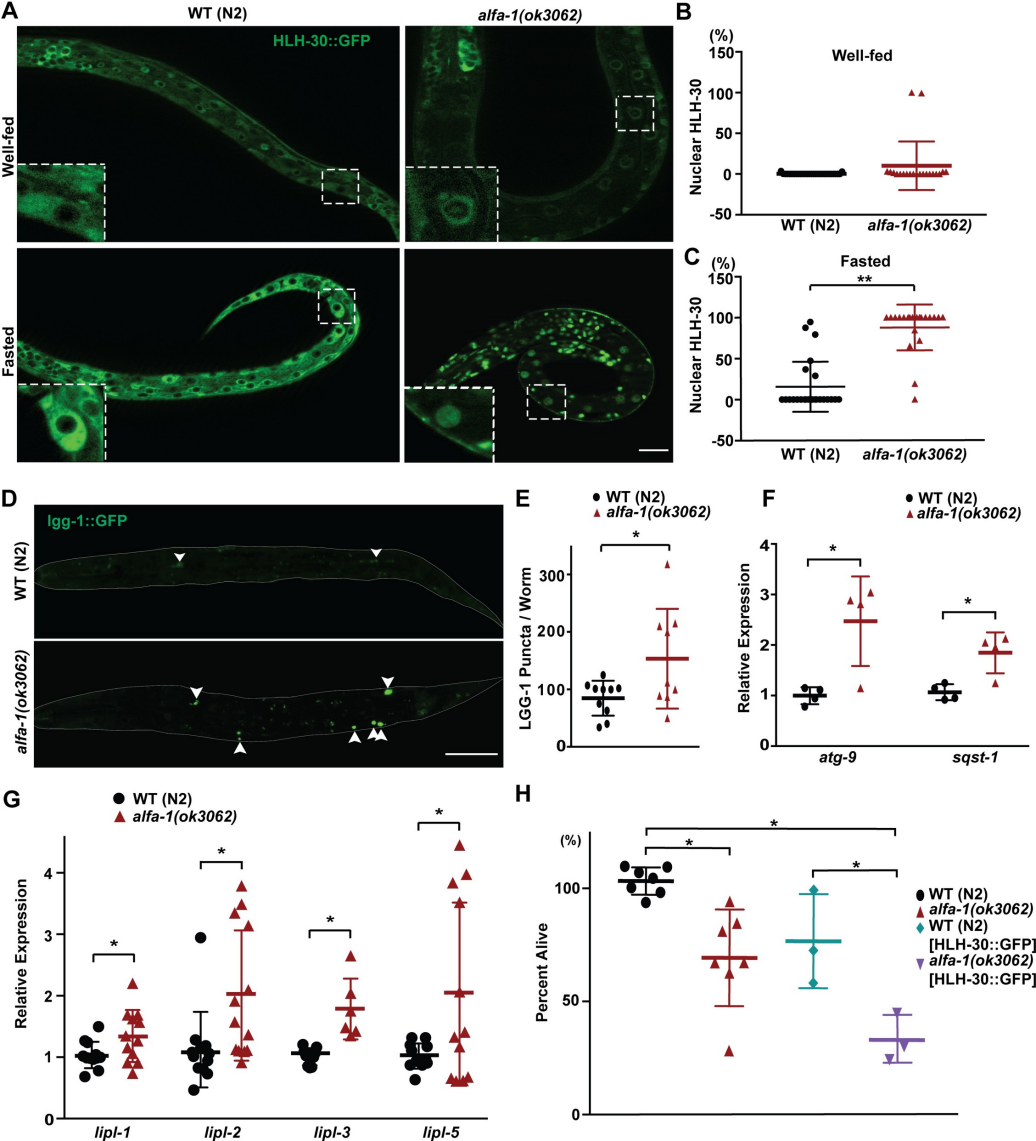
HLH-30/TFEB is abnormally activated in *alfa-1(ok3062)*. (A) Representative images of HLH-30::GFP expression patterns in wild-type or *alfa-1(ok3062) C*. *elegans* under well-fed (upper panels) or starvation (lower panels) conditions. Enlarged images of the boxed areas are shown in each panel. Wild-type worms at the L1 stage showed the signals in the cytoplasm of the cells, whereas *alfa-1(ok3062)* of the same stage showed the signals in the nuclei of the cells under starvation conditions. Well-fed L1 stage worms of both wild-type and *alfa-1(ok3062)* showed HLH-30::GFP in the cytoplasm. (B) Quantification of nuclear HLH-30::GFP in wild-type and *alfa-1(ok3062) C*. *elegans* under well-fed conditions. [*p* = 0.0799, n = 29 for wild-type and n = 21 for *alfa-1(ok3062) C. elegans*]. (C) Quantification of nuclear HLH-30::GFP in wild-type and *alfa-1(ok3062) C*. *elegans* under starvation conditions. [**p<0.0001, n = 24 for wild-type and *alfa-1(ok3062) C*. *elegans*]. (D) Representative images of LGG-1::GFP in the L1 worms starved for autophagy analysis. Worms are outlined with the gray line, and the white arrowhead indicates an autophagosome. (E) The number of LGG-1::GFP puncta per worm in the starved L1 worms. [*p<0.05, n = 10 for wild-type and n = 9 for *alfa-1(ok3062) C*. *elegans*]. (F) Relative expression levels of autophagy genes, including *atg-9* and *sqst-1*, as measured by RT-PCR, in starved L1-stage worms. [*p<0.05, n = 4 for all groups]. (G) Relative expression levels of four *lipase* genes, *lipl-1*, *lipl-2*, *lipl-3*, and *lipl-5*, as measured by RT-PCR, in starved L1-stage worms. [*p<0.05, n = 12 for *lipl-1*, *lipl-2*, and *lipl-5* and n = 6 for *lipl-3*]. (H) Percentage of HLH-30::GFP-expressing L1 worms that survived after 4 days of starvation. [*p<0.05, n = 7 for wild-type and *alfa-1(ok3062) C*. *elegans* and n = 3 for HLH-30::GFP-expressing wild-type and *alfa-1(ok3062) C*. *elegans*]. Distribution of data points is presented with mean ± SD. Scale bar: 20 μm.

TFEB, a basic helix-loop-helix leucine zipper transcription factor, is a master regulator of lysosome biogenesis, autophagy, and lipid metabolism [38–40]. HLH-30, the *C*. *elegans* orthologue of TFEB, has been reported to regulate gene expression involved in autophagy and lipolysis [41, 42]. As in mammalian cells, when adult *C*. *elegans* is starved, HLH-30 is translocated from the cytoplasm into the nucleus and turns on the expression of target genes promoting autophagy and lipolysis [41, 43]. Therefore, we utilized the HLH-30::GFP reporter to monitor the subcellular localization and activation status of the transcription factor. Interestingly, during starvation-induced L1 diapause, HLH-30::GFP was observed in the cytoplasm of all cells in N2 worms, suggesting that there is a mechanism to inhibit the nuclear translocation and activation of HLH-30 during L1 diapause in wild-type animals (Fig 2A and 2B). In contrast, *alfa-1(ok3062)* mutants in L1 diapause showed an HLH-30::GFP distribution in the nuclei of the cells, indicating that HLH-30 was abnormally activated in the absence of *alfa-1* (Fig 2A and 2C). The level of HLH-30::GFP expression appeared to be slightly increased, but not significantly statistically, in *alfa-1(ok3062)* when compared to that in wild-type N2 at the L1 stage under starvation conditions (S3C and S3D Fig).

Next, we confirmed that HLH-30 is functionally activated in *alfa-1(ok3062)* at L1 diapause. We first monitored autophagy using *C*. *elegans* LGG-1, the orthologue of mammalian LC3, labeled with GFP. Upon starvation, the distribution of LGG-1 changes from a diffuse pattern to a punctate pattern of autophagosomes in *C*. *elegans* [44, 45]. At L1 diapause, enlarged and increased numbers of autophagosomes, as marked by LGG-1::GFP, were observed in *alfa-1(ok3062)* mutants when compared to wild-type controls (Fig 2D and 2E), suggestive of heightened autophagic activity. Second, we measured the expression levels of the autophagy genes, *atg-9* and *sqst-1*, which are also HLH-30 target genes at the transcription level [42]. By RT-qPCR analysis, we found that the mRNA levels of *atg-9* and *sqst-1* were significantly higher in *alfa-1(ok3062)* at L1 diapause than in wild-type controls (Fig 2F), consistent with the notion that HLH-30 activation promotes the transcription of autophagy genes. Finally, we measured the expression level of four lipase genes, *lipl-1*, *lipl-2*, *lipl-3*, and *lipl-5*, that are known to be regulated by HLH-30 [41]. As expected, the levels of mRNA expressions for all the tested genes showed an upregulation in *alfa-1(ok3062)* at L1 diapause when compared to wild-type controls (Fig 2G). Taken together, these results support the concept that HLH-30 is abnormally activated in *alfa-1(ok3062)*.

### Abnormal activation of HLH-30/TFEB in *alfa-1(ok3062)* contributes to the decrease in lipids as well as survival at the L1 stage during starvation

We then asked how HLH-30 activities might contribute to the decreased survival phenotype at L1 diapause in *alfa-1(ok3062)* mutants. We first examined how HLH-30 overexpression influences survival at L1 diapause. Survival was decreased when HLH-30::GFP was overexpressed in wild-type N2 worms at L1 diapause, and the phenotype worsened when HLH-30::GFP was overexpressed in *alfa-1(ok3062)* (Fig 2H), consistent with the idea that the abnormal activation of HLH-30 in *alfa-1(ok3062)* causes a decrease in survival at L1 diapause.

**Fig 3:**
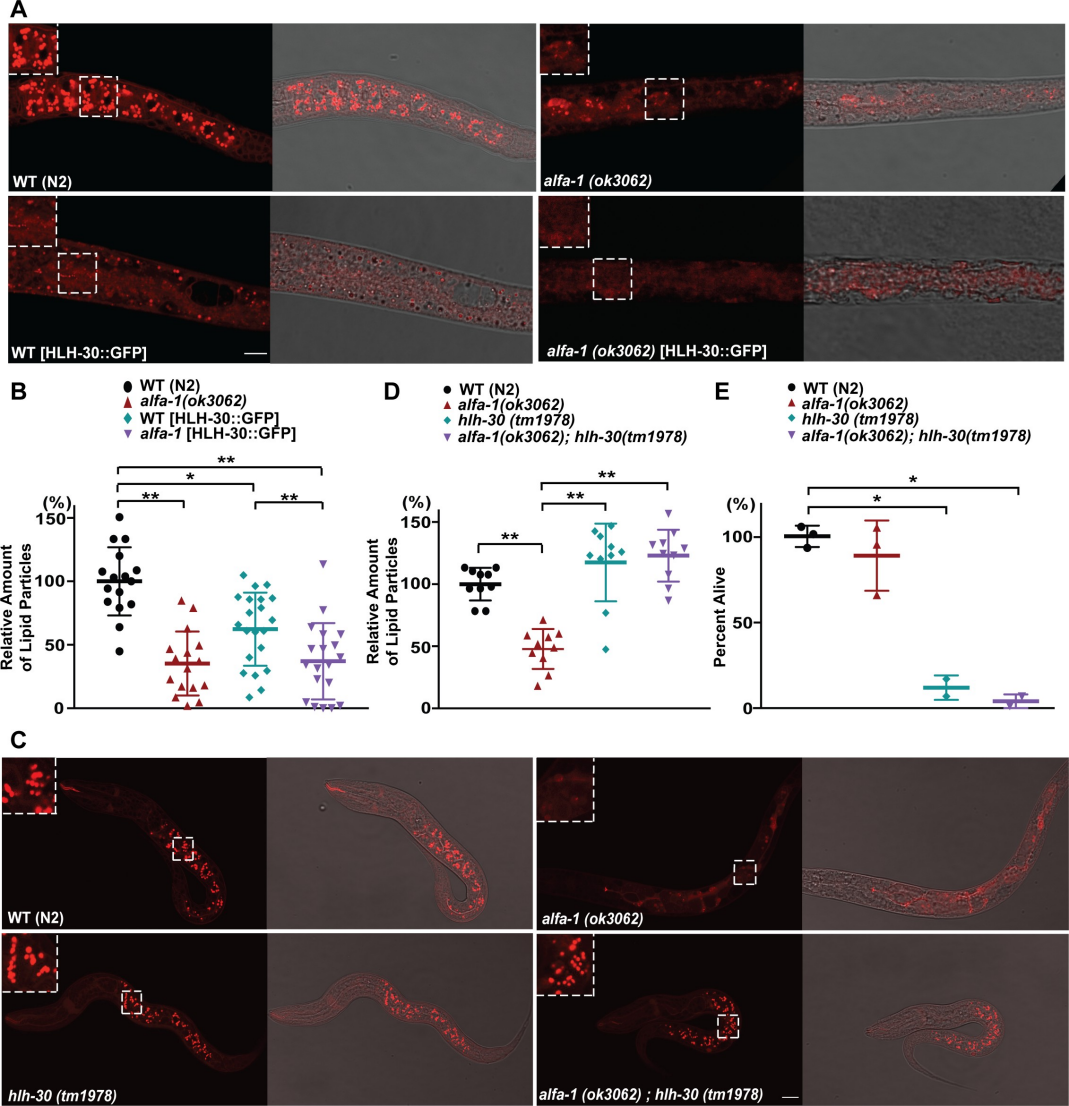
ALFA-1/C9orf72 regulates lipid metabolism through HLH-30/TFEB. (A) Representative images of Nile Red staining in N2, *alfa-1(ok3062)*, HLH-30::GFP-expressing wild-type and HLH-30::GFP-expressing *alfa-1(ok3062)* mutant *C*. *elegans* after 1 day of starvation. Enlarged images of boxed areas are shown in each panel. (B) Quantification of the lipid particles stained with Nile Red in HLH-30::GFP-expressing wild-type and *alfa-1(ok3062) C*. *elegans*. [*p<0.05, **p<0.0001, n = 16 for wild-type and *alfa-1(ok3062) C*. *elegans*; n = 21 for HLH-30-expressing wild-type and n = 26 for HLH-30-expressing *alfa-1(ok3062) C*. *elegans*]. (C) Representative images of Nile Red staining of N2, *alfa-1(ok3062)*, *hlh-30(tm1978)*, and *alfa-1(ok3062);hlh-30(tm1978)* within 8 hr after hatching in M9 buffer without food. Enlarged images of boxed areas are shown in each panel. (D) Quantification of the lipid particles stained with Nile Red in *hlh-30(tm1978)* and *alfa-1(ok3062);hlh-30(tm1978)*. [**p<0.0001, n = 10 for all groups]. (E) Percentage of L1 worms that survived, for N2, *alfa-1(ok3062)*, *hlh-30(tm1978)*, and *alfa-1(ok3062);hlh-30(tm1978)* after incubation in M9 buffer without food for 8 hr. [*p<0.05, n = 3 for wild-type and *alfa-1(ok3062) C*. *elegans* and n = 2 for *hlh-30(tm1978)* and *alfa-1(ok3062);hlh-30(tm1978)*]. Distribution of data points is presented with mean ± SD. Scale bar: 20 μm.

We then asked whether the overexpression of HLH-30 causes the decrease in lipid in lysosome-related organelles of *alfa-1(ok3062)* at L1 diapause. We determined whether HLH-30 is responsible for the digestion of lipids at L1 diapause by staining the worms with Nile Red. Similar to *alfa-1(ok3062)*, overexpression of HLH-30::GFP in wild-type N2 animals decreased the Nile Red staining at L1 diapause (Fig 3A and 3B), indicating that HLH-30 indeed promotes the digestion of the lipids. While overexpression of HLH-30::GFP in wild-type *C*. *elegans* decreased the Nile Red staining compared to the control, the level of Nile Red staining was still higher than in *alfa-1(ok3062)*, suggesting that overexpressed HLH-30::GFP was partially inhibited by ALFA-1 in wild-type worms. Indeed, loss of *alfa-1* in HLH-30::GFP overexpression *C*. *elegans* decreased the Nile Red staining signal to the level similar to that of *alfa-1(ok3062)* alone, suggesting that loss of *alfa-1* is sufficient to activate HLH-30 for lipid digestion.

To ascertain whether HLH-30 is responsible for the lipid-associated phenotypes in *alfa-1(ok3062)*, we examined the lipid levels using Nile Red staining in *C*. *elegans* carrying the loss-of-function allele *hlh-30(tm1978)*. In the absence of food, *hlh-30(tm1978) C*. *elegans* mutants preserved lipid particles, unlike *alfa-1(ok3062)* mutant, suggesting that HLH-30 is required for lipid digestion (Fig 3C and 3D). Interestingly, the level of Nile Red staining in *alfa-1(ok3062);hlh-30(tm1978)* double mutants was similar to those of wild-type worms, indicating that *alfa-1* regulates the lipid digestion through *hlh-30* (Fig 3C and 3D). Taken together, these data indicate that the *C*. *elegans* orthologue of C9orf72, ALFA-1, regulates the lipid metabolism through a mechanism dependent on the *C*. *elegans* orthologue of TFEB, HLH-30.

Finally, we tested if loss of *hlh-30* affects survival defects in *alfa-1(ok3062)*. However, the *hlh-30(tm1978)* mutant is viable for only a short period of time under conditions that lead to starvation-induced L1 diapause. Only a few *hlh-30(tm1978)* mutant animals survived at 8 hr after hatching without food (Fig 3E), and no worms survived at 48 hr after hatching (S3E Fig), indicating that HLH-30 is essential for survival during L1 diapause. Accordingly, no L1 larvae of the *alfa-1(ok3062);hlh-30(tm1978)* double mutation survived 48 hr without food (S3E Fig). These data suggest that HLH-30 is essential for lipid metabolism that is required for energy production and survival of L1 *C*. *elegans* during starvation.

Having established that *alfa-1* regulates lipid metabolism through HLH-30, we asked whether this regulation is related to the mTOR signaling. In mammals, TFEB, a homolog of HLH-30, is translocated into the nucleus when activated, and the nuclear translocation of TFEB is inhibited when TFEB is phosphorylated by the mTOR kinase [43, 46]. Therefore, we tested how an mTOR inhibitor, rapamycin, affects nuclear translocation of HLH-30. When wild-type *C*. *elegans* was treated with rapamycin, HLH-30::GFP was translocated to the nucleus at L1 diapause as expected (S4A and S4B Fig), suggesting that the *C*. *elegans* orthologue of TFEB, HLH-30, is also regulated by mTOR. Notably, rapamycin treatment in *alfa-1(ok3062)* resulted in a similar level of nuclear localization of HLH-30::GFP with *alfa-1(ok3062)* without the treatment, consistent with the notion that *alfa-1* and mTOR are in the same pathway in the regulation of HLH-30.

### C9orf72 regulates TFEB through Rag GTPases

**Fig 4:**
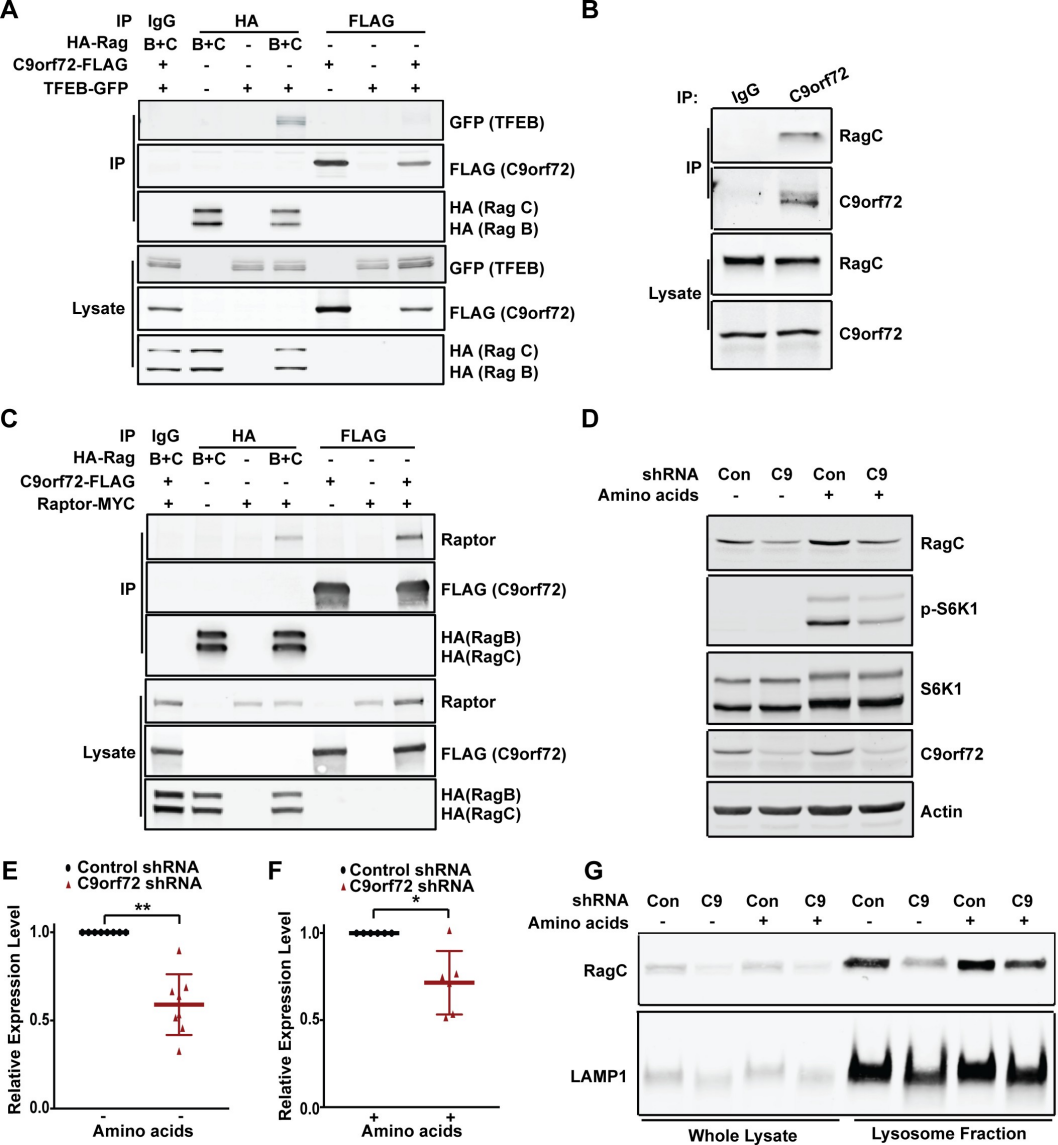
C9orf72 regulates TFEB through Rag GTPases. (A) Co-immunoprecipitation analysis of C9orf72-Flag and TFEB-GFP. HEK293 cells were transfected with the indicated plasmids, cell lysates were prepared at 48 hr post-transfection, and IgG, HA, or FLAG-tagged immunoprecipitates were analyzed by immunoblotting with anti-GFP, anti-FLAG, and anti-HA antibodies. HA-Rag immunoprecipitation was performed as a positive control. No GFP signal was detected in C9orf72-Flag immunoprecipitates. (B) Co-immunoprecipitation analysis of C9orf72 and RagC. RagC was detected in C9orf72 immunoprecipitates from HEK293 cells. (C) Co-immunoprecipitation analysis of C9orf72-Flag and myc-Raptor. HEK293 cells were transfected with the indicated plasmids, cell lysates were prepared at 48 hr post-transfection, and IgG, HA, or FLAG-tagged immunoprecipitates were analyzed by immunoblotting with anti-Raptor, anti-FLAG, and anti-HA antibodies. The HA-Rag immunoprecipitation and the detection of Raptor were performed as a positive control. (D) Representative images of immunoblotting for RagC levels in HEK293 cells expressing either control or C9orf72 shRNAs under amino acid starvation or stimulation conditions. Immunoblotting with anti-pS6K1(T389) and anti-S6K1 was performed to confirm the amino acid starvation or stimulation conditions. (E) Quantification of RagC levels under amino acid starvation conditions. Actin was used as the normalization control. [**p<0.0001, n = 8 for all groups]. (F) Quantification of RagC levels when the starved cells were stimulated with amino acids. Actin was used as the normalization control. [*p<0.05, n = 6 for all groups]. (G) Immunoblotting for RagC levels in the lysosomes of HEK293 cells expressing either control or C9orf72 shRNAs under amino acid starvation or stimulation conditions. Immunoblotting with anti-LAMP1 was performed to confirm the isolation of lysosomes. Distribution of data points is presented with mean ± SD.

To investigate the mechanisms through which C9orf72 regulates TFEB activities, we first asked whether C9orf72 directly associates with TFEB by performing co-immunoprecipitation analysis using C9orf72-Flag and TFEB-GFP expressed in HEK293 cells. However, no TFEB-GFP was detected in C9orf72-FLAG precipitates (Fig 4A), suggesting that C9orf72 and TFEB are not directly associated with each other and that another factor may mediate the functional association between these two proteins. As a positive control for the co-immunoprecipitation assay, we looked for an interaction between TFEB and the RagB/C heterodimer, two subunits of the Rag GTPase complex co-expressed in the cells, and observed this interaction as reported previously (Fig 4A) [47, 48].

In mammals, Rag GTPases function as a heterodimer consisting of two of four members of the family of Ras-related small GTP-binding proteins, RagA or RagB and RagC or RagD, with RagA and RagB being highly similar to each other and RagC and RagD also highly resembling each other [49, 50]. Since the Rag GTPases are known to recruit TFEB to the lysosome where mTOR phosphorylates TFEB and inhibits nuclear translocation [47, 48, 51], we asked whether the regulation of TFEB signaling by C9orf72 is mediated by Rag GTPases. To determine whether C9orf72 interacts with Rag GTPases, we performed co-immunoprecipitation in HEK293 cells expressing C9orf72-FLAG and HA-tagged RagB and RagC. When C9orf72 was pulled down with the Flag antibody, RagB and RagC were detected in the precipitates by western blotting, as compared to IgG control (S5A Fig). In the reciprocal immunoprecipitation, C9orf72-FLAG was detected in RagB/C-HA precipitates, but not in IgG control precipitates (S5B Fig). We also performed the co-immunoprecipitation analysis with all combinations of Rag GTPase complexes in the cells expressing C9orf72-FLAG and HA-Rags (S5C Fig). All combinations of Rag GTPase heterodimers were detected in C9orf72-FLAG immunoprecipitates, suggesting that C9orf72 is intrinsically capable of interacting with all forms of Rag GTPases. Furthermore, we confirmed the interaction between endogenous C9orf72 and RagC or RagA proteins (Fig 4B and S5D Fig). Consistent with the immunoprecipitation of overexpressed proteins, endogenous RagC or RagA was detected in the C9orf72 immunoprecipitates (Fig 4B and S5D Fig). These results established that C9orf72 interacts with the Rag GTPases under physiologically relevant conditions.

Since RagC binds Raptor, a component of the mTOR complex and a major regulator of mTORC1 [52], we asked whether C9orf72 is also associated with Raptor. Co-immunoprecipitation analysis was performed in HEK293 cells expressing C9orf72-FLAG and Raptor-MYC. When C9orf72 was pulled down with the Flag antibody, Raptor-MYC was detected in the precipitates by western blotting, as compared to the IgG control (Fig 4C), suggesting that C9orf72 is associated with the mTOR complex via its interaction with Rag GTPases and Raptor.

The Rag GTPases regulate the activation of mTORC1 upon amino acid stimulation [51, 52]. Since C9orf72 has been observed to influence mTORC1 signaling [22, 27], we asked whether C9orf72 regulates Rag GTPases, especially in response to amino acid stimulation. Interestingly, when HEK293 cells were grown under amino acid starvation conditions, there was a significant decrease in the level of RagC, as measured by western blotting, in the cells in which C9orf72 was knocked down with specific shRNAs, as compared to the cells treated with the control shRNAs (Fig 4D and 4E). More importantly, when the starved cells were stimulated with amino acids, the RagC levels were also significantly lower in the C9orf72-knockdown cells than in the control shRNA-treated cells (Fig 4D and 4F). In addition, the RagC level was also decreased in C9orf72 knockout MEF cells compared to wild-type cells under the same amino acid starvation and stimulation condition (S5E Fig), consistent with the results from HEK293 cells. We then asked whether the level of RagC on the lysosome is affected by the loss of C9orf72 under starvation or amino acid stimulation conditions. Lysosomes were isolated from HEK293 cells, and consistent with the changes in the whole lysates, the lysosomal level of RagC was significantly decreased in the C9orf72 knockdown cells under starvation and amino acid stimulation conditions, when compared to control cells (Fig 4G and S5F Fig). These data demonstrate that the level of RagC is dynamically regulated by C9orf72.

**Fig 5:**
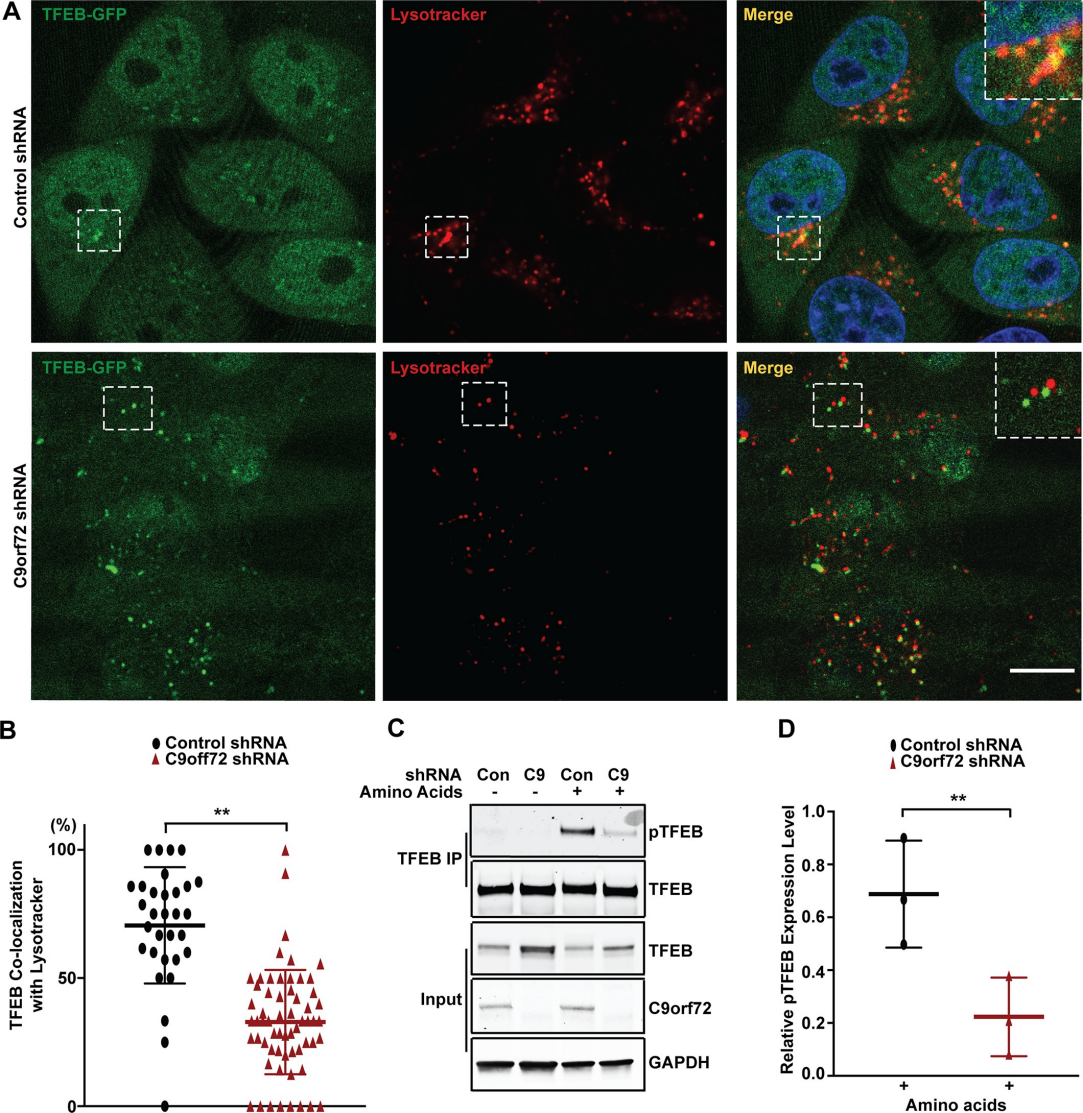
C9orf72 regulates TFEB localization on lysosomes. (A) Representative live-cell images of HeLa cells stably expressing TFEB-GFP and treated with control or C9orf72-specific shRNAs. Lysosomes were labeled with LysoTracker and the nucleus with NucBlue. Enlarged images of boxed areas are shown in the panel. Scale bar: 10 μm. (B) Quantification of TFEB-GFP localization in the lysosome. [**p<0.0001, n = 31 for control shRNA-expressing cells, and n = 61 for C9orf72-specific shRNA-expressing cells]. (C) Analysis of the levels of phosphorylated TFEB in HEK293 cells treated with C9orf72-specific or control shRNAs under amino acid starvation or stimulation conditions. Total TFEB was immunoprecipitated and phosphorylated TFEB was detected by western blotting in the precipitates. (D) Quantification of the levels of phosphorylated TFEB, which is normalized against that of total TFEB. [**p<0.0001, n = 3 for all groups]. Distribution of data points is presented with mean ± SD.

**Fig 6:**
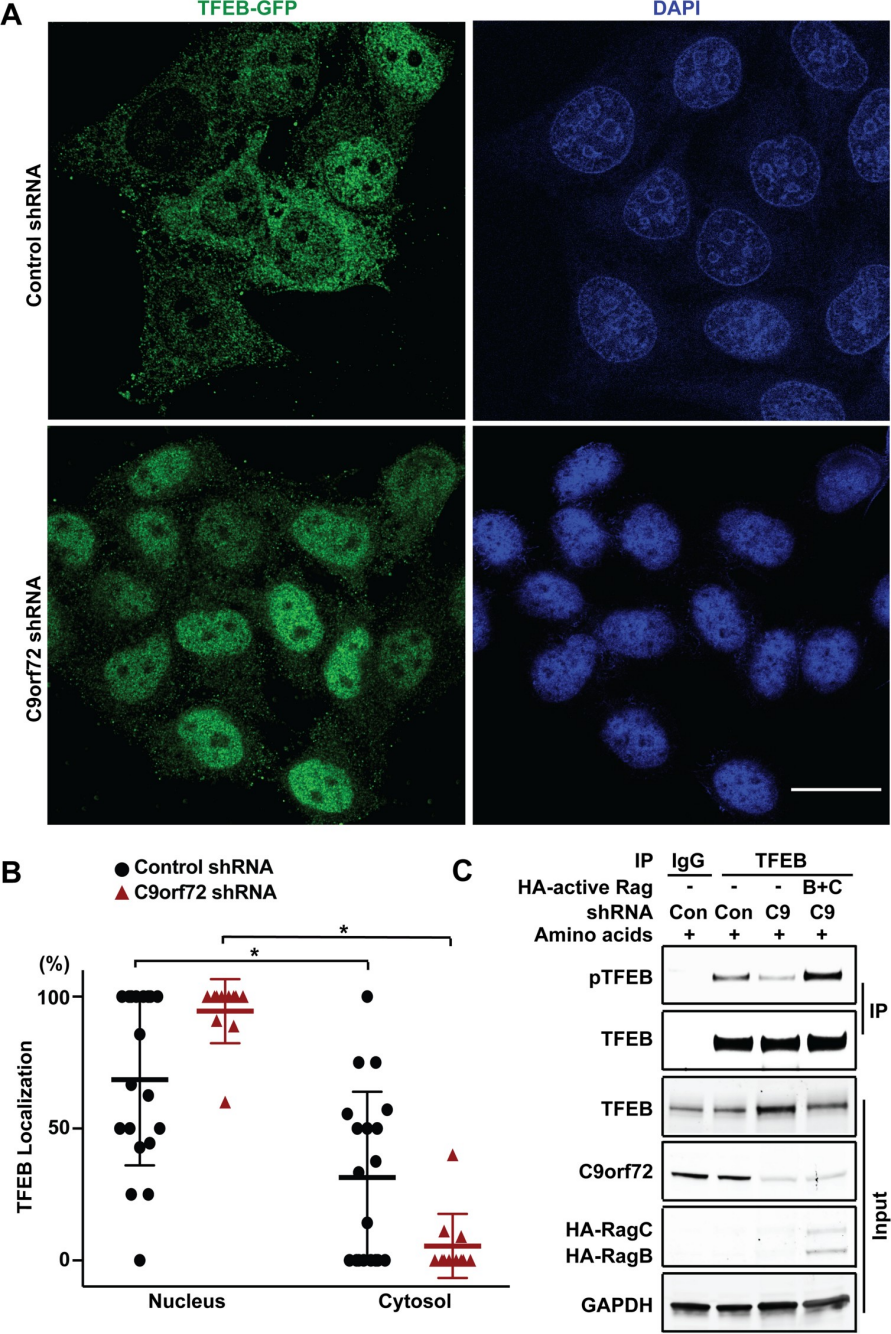
C9orf72 regulates TFEB translocation from the nucleus to the cytoplasm upon amino acid stimulation. (A) Representative immunostaining images of HeLa cells stably expressing TFEB-GFP, with C9orf72 or control shRNA knockdown, after amino acid stimulation for 10 min following a period of 50 min of amino acid starvation. Immunostaining with anti-GFP and an Alexa488-conjugated secondary antibody was performed together with DAPI staining. Scale bar: 20 μm. (B) Quantification of TFEB-GFP subcellular localization in (a). The numbers of cells carrying nuclear or cytoplasmic TFEB-GFP were quantified. [*p<0.05, n = 19 for control shRNA-expressing cells and n = 11 for C9orf72-specific shRNA-expressing cells]. Distribution of data points is presented with mean ± SD. (C) Decreased phosphorylated TFEB level was rescued by expression of an active forms of Rag GTPases when the starved cells were stimulated with amino acids. Total TFEB was immunoprecipitated and phosphorylated TFEB was detected by western blotting in the precipitates.

Since Rag GTPases mediate amino acid-dependent recruitment of TFEB to lysosomes, we asked whether TFEB subcellular localization is changed in C9orf72-deficient cells under amino acid stimulation. We monitored the lysosomal localization of TFEB using the TFEB-GFP reporter and LysoTracker to label lysosomes. Upon amino acid stimulation, TFEB-GFP was co-localized with LysoTracker in the control shRNA-treated cells (Fig 5A and 5B), consistent with the previous reports [47, 48]. Importantly, the co-localization of TFEB with the LysoTracker in response to amino acid stimulation was decreased in C9orf72 shRNA-treated cells when compared to control cells (Fig 5A and 5B). Next, we asked whether the phosphorylation of TFEB, which determines its nuclear localization, is also altered. Since the amount of phosphorylated TFEB was too low to be detected reliably in total cell lysates from HEK293 cells, we used immunoprecipitation to pull down total TFEB and then analyzed the level of phosphorylated TFEB. As expected, TFEB phosphorylation was increased upon amino acid stimulation; however, the degree of TFEB phosphorylation was lower in the C9orf72 shRNA-treated cells than in control shRNA-treated cells, consistent with the impairment in Rag GTPase functions and mTOR signaling, as indicated by reduced levels of RagC and phosphorylated S6K1, respectively, in the C9orf72-deficient cells (Fig 5C and 5D). Consistent with the decrease of phosphorylated TFEB, the TFEB localization in the nucleus was increased in C9orf72 shRNA-treated cells over control cells after amino acid stimulation (Fig 6A and 6B). Finally, to confirm that the effects of C9orf72 on TFEB was mediated by Rag GTPases, we used a constitutively active form of Rag GTPases to test if it can rescue the phenotype in C9orf72-deficient cells. Because the Rag proteins function as heterodimers in which the active complex consists of GTP-bound RagA or B complexed with GDP-bound RagC or D, we overexpressed the GTP-bound form of RagB with GDP-bound form of RagC to rescue the decrease of phosphorylated TFEB in C9orf72-deficient cells [53]. For the GTP-bound form of RagB, the RagB^Q99L^ mutant that lacks GTPase activity was used, and for the GDP-bound form of RagC, that RagC^S75N^ mutant that has deficient affinity for nucleotides was used. As expected, the decrease of phosphorylated TFEB in C9orf72-deficient cells was rescued by overexpression of the active form of Rag GTPases, confirming that C9orf72 regulates TFEB through Rag GTPases (Fig 6C). Taken together, these results support a model in which C9orf72 regulates TFEB inactivation through Rag GTPases under conditions of amino acid stimulation (S5 Fig).

## Discussion

The present study has revealed a conserved role for C9orf72 in the regulation of TFEB, a master transcription factor for autophagy, lysosome biogenesis, and lipid metabolism. We found that in *C*. *elegans*, loss of the C9orf72 orthologue, ALFA-1, leads to nuclear translocation and abnormal activation of HLH-30/TFEB under nutrient stress. We also uncovered that the same regulation of TFEB by C9orf72 exists in human cells and is mediated by the Rag GTPases, the key player at the lysosome surface in mTOR signaling.

These results are consistent with the emerging notion that C9orf72 is an important regulator of metabolic homeostasis, particularly in response to nutrient stress. Identification of Rag GTPases as an interactive partner and effector of C9orf72 illustrates a mechanism for the regulation of mTOR and TFEB signaling and downstream metabolic processes. In response to amino acid signals, Rag GTPases bind to mTORC1 and promotes the translocation of mTORC1 to the lysosome surface [54], where mTORC1 phosphorylates TFEB and inhibits the latter’s nuclear translocation and activation [48]. We found that C9orf72 forms a complex with Rag GTPases and that a deficiency in C9orf72 leads to a decrease in the level of RagC under conditions of nutrient stress. The deficits in Rag GTPase functions can impair the mTOR signaling response to amino acid signals and in turn lead to abnormal activation of TFEB, which we indeed observed in C9orf72-deficient cells. The regulation of TFEB by C9orf72 appears to be an ancient pathway, given the similarity to the ALFA-1−HLH-30 pathway in *C*. *elegans*. Overall, C9orf72 acts as a brake in the dynamic regulation of nutrient-dependent mTOR and TFEB signaling; its deficiency can cause overactivation of metabolic systems and negatively affect fitness during evolution, potentially explaining the survival phenotype of *C*. *elegans* lacking ALFA-1/C9orf72 during L1 diapause under nutrient stress. Consistently, in various cellular and animal models, loss of C9orf72 does not cause an overt phenotype under normal conditions but leads to more apparent deficits under stress conditions [18, 31, 55, 56]. Notably, while this manuscript was in review, an independent study reported that C9orf72 associates with Rag GTPases and regulates autophagosomal and lysosomal biogenesis [57], consistent with the main observations of the present study.

Nutrient sensing and metabolic processes such as the autophagic and lysosomal degradation are essential for neuronal health, and their alteration is an increasingly recognized feature in aging-related neurodegenerative diseases [58–60]. For instance, there is a multitude of ALS genes with functions related to autophagy [61], suggesting that the autophagic and metabolic defects are a common theme for ALS and related neurodegeneration. Taken together, our findings suggest that the C9orf72 protein plays a role in key nutrient-sensing and metabolic processes, and its dysregulation may contribute to age-dependent neurodegenerative diseases.

## Materials and methods

### C. elegans

All *C*. *elegans* strains were maintained at 20°C under standard conditions. *E*. *coli* OP50 was used for feeding, and the N2 Bristol strain was used as the wild-type control. Mutant strains were obtained from the *Caenorhabditis* Genetics Center, including RB2260 *alfa-1(ok3062)* II, CB1370 *daf-2(e1370)* III, CF1038 *daf-16(mu86)* I, JIN1375 *hlh-30(tm1978) IV*, and DA2123 *adIs2122[lgg-1*::*GFP rol-6(df)]*. OP433 *wgIs433 [hlh-30*::*TY1*::*EGFP*::*3xFLAG + unc-119(+)]* was kindly provided by Dr. Eyleen J. O'Rourke at University of Virginia. All the mutant strains were backcrossed with the N2 strain at least four times. The new strains generated by crossing in this study include: IW450 *alfa-1(ok3062) II; daf-2(e1370) III*, IW451 *alfa-1(ok3062)II;daf-16(mu86) I*, IW762 *alfa-1(ok3062)II;wgIs433 [hlh-30*::*TY1*::*EGFP*::*3xFLAG + unc-119(+)]*, IW734 *alfa-1(ok3062)II;hlh-30(tm1978) IV*, and IW366 *adIs2122[lgg-1*::*GFP rol-6(df)]; alfa-1(ok3062)II*.

### ALFA-1 antibody generation and validation

A synthetic peptide (RAEILQPDISEFIYM) corresponding to the C-terminal region of ALFA-1 was synthesized and used to immunize rabbits. The resulting polyclonal antibodies were purified using affinity chromatography (ThermoFisher). The specificity of the ALFA-1 antibody was validated by pre-incubating the antibody with the immunizing peptide for 30 min at room temperature prior to immunoblot analysis.

### *C*. *elegans* immunoblot analysis

For ALFA-1 immunoblotting, mixed-stage N2 and *alfa-1(ok3062)* worms were harvested in M9 buffer from NGM plates and washed five times in M9 buffer to remove the *E*. *coli* OP50. For HLH-30::GFP immunoblotting, eggs of *wgIs433* and *alfa-1(ok3062);wgIs433* were prepared, and cultured in M9 Buffer at 20°C. Then the L1 larva was collected 24 hours after hatching. The worms were lysed in modified RIPA buffer (50 mM Tris, pH 6.8, with 150 mM NaCl, 0.5% SDS, 0.5% Sarkosyl, 0.5% NP40, 20 mM EDTA, and Roche cOmplete, EDTA-free protease inhibitor cocktails), freeze-thawed three times, and sonicated using a Diagenode Bioruptor for 15 min (high setting, 30-sec pulse). The resulting lysates were then centrifuged at 16,000 x g for 10 min at 4°C to pellet worm debris. Protein concentrations were determined using the bicinchoninic acid assay (ThermoFisher) and 50 μg of protein was separated by SDS-PAGE. The resulting protein was transferred to a nitrocellulose membrane using the Mini Trans-Blot Turbo transfer system (Bio-Rad). The membrane was incubated with the primary antibody overnight at 4°C before being incubated with the anti-rabbit secondary antibody and visualized using an Odyssey imaging system (Li-cor).

### L1 diapause survival assay

Gravid adult worms were collected in 3.5 ml of M9 buffer and treated with 1 ml of bleach solution and 0.5 ml of 5 N NaOH to extract the eggs. Eggs were washed at least three times before culturing the worms. After washing, the egg solution was prepared at a concentration of 1–2 eggs/μl of M9 buffer in a 15-ml conical tube and was cultured in a shaking incubator at 20°C for the indicated time. Days were counted from egg preparation: day 1 was 24 hr after hatching, day 2 was 48 hr, and so on. At each time point, a 100-μl aliquot (approximately 100–200 worms) from each sample tube was plated on each of three plates seeded with *E*. *coli* OP50. The tube was returned to the shaking incubator for further days of starvation. Plated worms were allowed to grow for 3 days, and the number of worms that reached the L4 or adult stage was counted from each plate. The numbers from three plates were averaged and recorded. The number at hatching was used as the total number of worms to be analyzed and the denominator to calculate the percentage of worms recovering after 3 days of starvation.

### *C*. *elegans* transcriptome profiling

Mixed-stage *alfa-1(ok3062)* and matched N2 *C*. *elegans* were bleached, and the resulting eggs seeded on to NGM plates and allowed to incubate at 20°C. Approximately 48 hr after seeding, L3/L4 worms were collected in M9 buffer and rinsed five times to remove any residual OP50 bacteria. The worms were then equally divided into 1.5 ml-tubes. Control (non-starved) samples were immediately centrifuged, and the resulting worm pellets were frozen at -80°C. The remaining worm samples were allowed to incubate in M9 buffer at room temperature for 6 hr with gentle mixing. After 6 hr, the samples were treated in an identical manner to the controls and stored -80°C until further downstream processing. In total, four samples per strain per condition were used for analysis. Total RNA from all samples was extracted using the RNeasy MiniPrep Kit (Qiagen) and analyzed using Affymetrix *C*. *elegans* microarrays as previously described [62]. The microarray data were also analyzed through the use of Ingenuity Pathways Analysis (IPA) (Ingenuity Systems, www.Ingenuity.com). The differentially regulated *C*. *elegans* genes that were associated with biological functions in Ingenuity’s Knowledge Base were analyzed to identify the biological functions that were most significant to the data set. Microarray data can be found at the Gene Expression Omnibus repository (accession #: GSE137355).

### Nile red staining

Nile Red powder (ThermoFisher, Cat. # N1142) was dissolved in acetone at 1 mg/ml, diluted in M9 buffer at 5 μg/ml, and added to 200 μl of Nile Red solution in M9 on top of nematode growth medium (NGM) seeded with OP50 in a 6-cm plate. Worms were placed on these plates and were cultured until they became gravid adults. Eggs were prepared as described for the survival assay above, and Nile Red was added at the same concentration (0.1 μg/mL) to the egg solution. For starvation conditions, eggs were cultured in M9 buffer. Nile Red images of 100 mM levamisole-treated worms on 2% agarose pads were taken under the confocal microscope approximately 24 hr after hatching, and then analyzed using Image J software. For well-fed larva, eggs were cultured in M9 buffer supplemented with *E*. *coli* OP50, and images were taken after 16 hr of culture at the L1 stage. For young adults, eggs were placed on an NGM plate seeded with OP50, to which was added the Nile Red solution. Eggs were cultured in the 20°C incubator for approximately 54 hr, until the worms reached the young adult stage, and then imaged. For the quantification of Nile Red staining, particle analysis was performed using Image J, and the number of particles per worm was counted and compared to those in wild-type controls.

### HLH-30::GFP reporter assay

The L1 larva from HLH-30::GFP reporter-expressing worms were prepared and cultured for approximately 24 hr in M9 buffer for starvation. The GFP images of HLH-30::GFP-expressing worms that had been paralyzed by mixing 100 mM levamisole with equal volume of worm slurries in M9 on the 2% agarose pads were then taken using a Zeiss ApoTome. For the well-fed conditions, the eggs were cultured in M9 buffer supplemented with OP50 for 16 hr and then imaged. The numbers of nuclear GFP-expressing cells and of cytoplasmic GFP-expressing cells per worm were counted for the quantification.

### Lifespan analysis

Twenty gravid adult worms per group were placed on NGM plates with OP50 to collect approximately 300 eggs; the adult worms were removed after 2 hr. The eggs were then cultured approximately 54 hr until they reached the L4 stage. The L4 worms were transferred to a new plate, and the next day they were considered to be day 1 adults as they started to lay eggs. From day1, the dead worms were counted on every other day, until all the worms were dead. The worms were transferred to the new plates every other day until they either stopped laying eggs, to avoid counting offspring, or they consumed all the *E*. *coli*.

### Quantitative real-time PCR

Total RNA was isolated with an RNeasy Plus Mini kit from L1 worms under starvation conditions, and cDNAs were synthesized with the QuantiTect reverse transcription kit (Qiagen). Primer sequences for qPCR are listed in S1 Table. RT-qPCRs were performed on a CFX96 Real-Time System thermal cycler (BioRad) with iQ SYBER Green PCR mix (BioRad).

### *C*. *elegans* autophagy analysis

Starved L1 larva expressing the LGG-1::GFP reporter were collected as described above. The worms were placed on a 2% agarose pad with levamisole and imaged under an SP8 confocal microscope (Leica). The images were then analyzed by particle analysis in Image J to count the autophagosome puncta and to measure the size of autophagosomes. The number of autophagosomes per worm was averaged and the size distribution analyzed.

### DNA plasmids and antibodies

C9orf72-Flag and C9orf72 shRNA constructs were described previously [27]. GFP-TFEB (38119, Shawn Ferguson, Yale University), pRK5-HA GST RagB (19301, David Sabatini, Whitehead Institute), pRK5-HA GST RagC (19304, David Sabatini, Whitehead Institute), myc-Raptor (1859, David Sabatini, Whitehead Institute), pRK5-HA GST RagB 99L (19303, David Sabatini, Whitehead Institute), and pRK5-HA GST RagC 75N (19305, David Sabatini, Whitehead Institute) were obtained from Addgene.

Antibodies used in the human cell studies include the following: mouse anti-Flag (Sigma), mouse anti-GFP, rabbit anti-GAPDH (ThermoFisher), mouse anti-actin (Santa Cruz), mouse anti-C9orf72 (Bio-Rad), mouse anti-C9orf72 (Proteintech), mouse anti-HA (Bethyl Lab), rabbit anti p-70S6K, rabbit anti p-p70S6K, rabbit anti-RagA, rabbit anti-RagC, rabbit anti-Raptor, rabbit anti-LAMP1, rabbit anti-DYKDDDDK, rabbit anti-pTFEB, rabbit anti-TFEB (Cell Signaling), mouse anti-TFEB (Mybiosources), and rabbit anti-GFP (Abcam).

### Cell culture and DNA transfection

All cells were maintained in DMEM supplemented with 10% FBS in a 5% CO_2_, 37 C incubator. HEK293 cells stably expressing control or C9orf72 shRNA were cultured in DMEM supplemented with 10% FBS and 2 μM puromycin. HeLa cells stably expressing TFEB-GFP were cultured in DMEM with 10% FBS supplemented with 10% FBS and 400 μM G418.

For co-transfection experiments, 2.2 X 10^6^ cells were plated in PEI-coated 10-cm culture dishes. The next morning, the cells were transfected in OMEM using Lipofectamine 2000 (ThermoFisher) according to the manufacturer’s instructions. Transfected plasmid DNA amounts were: 2 μg of C9orf72-Flag, 100 ng of pRK5-HA GST RagB or RagC, 50 ng of myc-Raptor, and 1 μg of TFEB-GFP. The total amount of plasmid DNA in each transfection was normalized to 2.5 μg with an empty pRK5-myc vector. Five hours after transfection, the OMEM was replaced with DMEM supplemented with 10% FBS. At 48 hr post-transfection, the cells were harvested for co-immunoprecipitation analysis.

### Amino acid starvation and stimulation of cells

Cells (0.8 X 10^6^) were plated in 60-mm plates and cultured for 72 hr prior to starvation treatment. Then the cells were rinsed two times with amino acid-free RPMI and incubated in the same medium (MyBiosources, Cat. # MBS652918) for 50 min, then rinsed and stimulated with complete RPMI 1640 (Gibco, Cat. # 11875) for 10–15 min.

### Immunoblot analysis

Cells were collected in lysis buffer (20 mM Tris-HCl, pH 7.5, with 137 mM NaCl, 1% NP-40, 20 mM EDTA, Roche cOmplete, EDTA-free protease inhibitor cocktails, and Roche PhosSTOP phosphatase inhibitor cocktails) and sonicated using a Diagenode Bioruptor for 15 min (high setting, 30-sec pulse). Soluble fractions of the lysates were collected by centrifuging at 16,000 x g for 10 min at 4°C, and protein concentrations were determined using the bicinchoninic acid assay (ThermoFisher). Fifty micrograms of protein per lane were resolved on SDS-PAGE gels and then transferred to nitrocellulose membranes using a Trans-blot Turbo transfer system (Bio-Rad). Western blotting was performed with an appropriate primary antibody and IRDye 800CW or 680RD secondary antibody, and the signal was detected using an Odyssey imaging system (Li-Cor).

### Immunoprecipitation

Cells were rinsed twice with PBS and lysed in NP-40 lysis buffer (20 mM Tris-HCl, 150 mM NaCl, 1% NP-40 and 2 mM EDTA), then sonicated for 15 min on ice. The soluble fraction of the lysate was collected by centrifugation at 16,000 x g for 10 min prior to immunoprecipitation as described above. For immunoprecipitations, the primary antibodies were added to the lysate and incubated on a rotator overnight at 4°C. The immune complexes were then pulled down with protein A/G magnetic beads (ThermoFisher, Cat. # 88803) and washed three times with washing buffer according to the manufacturer’s manual. The immunoprecipitates were eluted by incubating the beads with SDS-PAGE loading dye for 10 min at room temperature and boiled for 10 min at 95°C prior to being loaded on the SDS-PAGE gel. Western blotting was performed as described above. For immunoprecipitation of endogenous proteins, western blotting was performed with the quick western kit (Li-Cor, Cat. # 926–69100) according to the manufacturer’s instructions.

### Live cell imaging and immunostaining

For live-cell imaging, 3 X 10^5^ HeLa cells stably expressing TFEB-GFP and control or C9orf72 shRNA were grown on glass-bottomed 35-mm dishes for 72 hr, then rinsed twice with and incubated with amino acid-free RPMI medium for 50 min. In the middle of the starvation treatment, LysoTracker Red DND-99 (ThermoFisher Scientific, Cat. # L7528) at a final concentration of 50 nM and a drop of NucBlue Live Cell Stain ReadyProbes (ThermoFisher Scientific, Cat. # R37605) were added to the medium to stain the lysosomes and nuclei. The cells were then washed and incubated with complete RPMI 1640 medium (Gibco, Cat. # 11875). The cells were imaged immediately and within 15 min after the complete medium was added using an SP8 confocal microscope (Leica).

For immunostaining, 1 X 10^5^ HeLa cells expressing TFEB-GFP and control or C9orf72 shRNA were plated on glass coverslips in 12-well tissue culture plates and cultured for 72 hr. The cells were starved and stimulated by amino acid as described above. After amino acid stimulation for 10 min, the cells were washed twice with PBS, fixed with cold methanol for 5 min, washed with PBC three times, and incubated with blocking buffer (5% normal donkey serum in PBS). Rabbit anti-GFP (diluted in blocking buffer 1:1,000) was incubated with the cells overnight at 4°C, and the cells were then washed with PBS three times. Alexa 488-conjugated anti-rabbit secondary antibody was added at a 1:1000 dilution, and the cells were incubated in the dark for 2 hr at room temperature, then washed with PBS three times. Coverslips were mounted with Prolong Gold with DAPI (ThermoFisher, Cat. # P36931) and imaged with a 63X objective lens using an SP8 confocal microscope (Leica).

### Quantitation and statistical analysis

All quantitation and statistical tests were performed using ImageJ and GraphPad Prism software (Version 7.0). The p-values were obtained using unpaired Student’s *t*-tests, unless otherwise noted in the figure legends.

## Supporting information

S1 Fig Genetic interaction analysis of *alfa-1*(*ok3062*) with *daf-2*(*e1370*) or *daf-16*(*mu86*). (A) Brood size analysis of *alfa-1(ok3062)*. The total numbers of progeny were counted for the N2 and mutant strains. Distribution of data points are presented with mean ± SD. [*p* = 0.7679, and n = 10 for all groups) (B) The percentages of wild-type, *alfa-1(ok3062)*, *daf-2(e1370)*, and *alfa-1(ok3062);daf-2(e1370) C*. *elegans* surviving to adulthood after incubation of L1 worms in M9 buffer without food. The survival of *alfa-1(ok3062)* and *alfa-1(ok3062);daf-2(e1370)* was significantly decreased compared to wild-type worms [*p*<0.0001], while the survival of *daf-2(e1370)* was significantly increased [*p*<0.0001]. The survival of *alfa-1(ok3062);daf-2(e1370)* was similar to that of *alfa-1(ok3062)*. [*p* = 0.3305, n = 4502 for N2, n = 4151 for *alfa-1(ok3062)*, n = 2523 for *daf-2(e1370)*, and n = 3257 for *alfa-1(ok3062);daf-2(e1370)*]. Log-rank (Mantel-Cox) test was used and data are presented as means. (C) The percentages of wild-type, *alfa-1(ok3062)*, *daf-16(mu86)*, and *daf-16(mu86);alfa-1(ok3062) C*. *elegans* surviving to adulthood after incubation of L1 worms in M9 buffer without food. The survival of *daf-16(mu86)* and *daf-16(mu86);alfa-1(ok3062)* was significantly decreased compared to wild-type worms [*p*<0.0001], and the survival of *daf-16(mu86);alfa-1(ok3062)* was significantly decreased compared to *alfa-1(ok3062)*. [*p*<0.0001, n = 1140 for *daf-16(mu86)* and n = 352 for *daf-16(mu86);alfa-1(ok3062)*]. Log-rank (Mantel-Cox) test was used and data are presented as means. (D) Lifespan analysis of *alfa-1(ok3062)* with *daf-2(e1370)* or *daf-16(mu86)* under normal conditions. The lifespan of *alfa-1(ok3062)* was not changed compared to N2 [*p* = 0.3428]. The lifespan of *daf-2(e1370)* was significantly increased, while the lifespan of *daf-16(mu86)* was significantly decreased compared to N2 [*p*<0.0001]. The lifespan of *alfa-1(ok3062);daf-2(e1370)* and *daf-16(mu86);alfa-1(ok3062)* was similar to that of *daf-2(e1370)* [*p* = 0.3675] and *daf-16(mu86)* [*p* = 0.7289], respectively. [n = 508 for N2, n = 501 for *alfa-1(ok3062)*, n = 120 for *daf-2(e1370)*, n = 185 for *daf-16(mu86)*, n = 77 for *alfa-1(ok3062);daf-2(e1370)*, and n = 109 for *daf-16(mu86);alfa-1(ok3062)*]. Log-rank (Mantel-Cox) test was used and data are presented as means.
(TIF)

S2 Fig Transcriptome analysis of WT and *alfa-1*(*ok3062*) *C*. *elegans*. (A) The schematic diagram for the transcriptome analysis of wild-type and *alfa-1(ok3062) C*. *elegans*. N2 and *alfa-1(ok3062)* were synchronized by bleaching and cultured until they reach to L3/L4 stage in the well-fed condition, then the worms equally divided into two groups. One group (+ Food) was frozen for further analysis and the other group (- Food) was starved for 6 hr. Then, the total RNAs were extracted and subjected to the transcriptome profiling using microarrays. (B) The top five molecular and cellular functions of differentially expressed genes in well-fed wild-type and *alfa-1(ok3062)* mutants. Rankings are given from top to bottom. Each dot represents a gene with a *p*-value. (C) Percentages of molecules found in (b) related to each molecular and cellular function. (D) The top five molecular and cellular functions of differentially expressed genes in fasted wild-type and *alfa-1(ok3062) C*. *elegans*. Rankings are given from top to bottom. Each dot represents a gene with a *p*-value. (E) Percentages of the molecules found in (d) related to each molecular and cellular function.
(TIF)

S3 Fig Characterization of *C*. *elegans* lacking the orthologue of C9orf72, *alfa-1*. (A) Representative image of Nile Red staining patterns in well-fed adult worms. Both Nile Red staining patterns (upper panels) and DIC images (lower panels) are shown. Scale bar: 100 μm. (B) Quantification of the lipid particles stained with Nile Red in adult wild-type and *alfa-1(ok3062) C*. *elegans* under well-fed conditions. [*p* = 0.3140, n = 5 for all groups]. (C) Immunoblotting of HLH-30::GFP in wild-type and *alfa-1(ok3062)* worms under starvation conditions. (D) Quantification of HLH-30::GFP levels in (C) with actin as the control. [*p* = 0.5788, n = 3 for all groups]. (I) The percentages of worms surviving to adulthood after incubation of L1 worms in M9 buffer without food for 2 days for N2, *alfa-1(ok3062)*, *hlh-30(tm1978)*, and *alfa-1(ok3062);hlh-30(tm1978)* [*p<0.05, and n = 3 for all groups]. Distribution of data points is presented with mean ± SD.
(TIF)

S4 Fig Inhibition of mTOR by rapamycin enhances HLH-30::GFP translocation into the nucleus in *C*. *elegans*. (A) Representative images of HLH-30::GFP in wild-type and *alfa-1(ok3062)* L1 *C*. *elegans* that were treated with DMSO or rapamycin in the absence of food. Enlarged images of boxed areas are shown in each panel. (B) Percentages of nuclear HLH-30::GFP in starved L1 with or without rapamycin. [*p<0.05, **p<0.0001, n = 10 for DMSO groups and n = 15 for rapamycin groups]. Distribution of data points is presented with mean ± SD. Scale bar: 20 μm.
(TIF)

S5 Fig The interaction between C9orf72 and Rag GTPases. (A) Co-immunoprecipitation analysis of C9orf72-Flag and HA-Rag. HEK293 cells were transfected with the indicated plasmids, cell lysates were prepared at 48 hr post-transfection, and IgG or FLAG-tagged immunoprecipitates were analyzed by immunoblotting with anti-FLAG and anti-HA antibodies. (B) Reciprocal co-immunoprecipitation analysis of HA-Rag and C9orf72-Flag. Cell lysates were prepared as described above, and IgG or HA-tagged immunoprecipitates were analyzed by immunoblotting with anti-FLAG and anti-HA antibodies. (C) Co-immunoprecipitation analysis of C9orf72-Flag and different HA-Rag isoforms. HEK293 cells were transfected with the indicated plasmids, cell lysates were prepared as described above, and IgG or FLAG-tagged immunoprecipitates were analyzed by immunoblotting with anti-FLAG and anti-HA antibodies. (D) Co-immunoprecipitation analysis of C9orf72 and RagA indicates that RagA was detected in C9orf72 immunoprecipitates from HEK293 cells. (E) Immunoblotting analysis showed that RagC protein levels were decreased in *C9orf72*^*-/-*^ MEF cells under amino acid starvation or stimulation conditions. (F) Quantification of RagC poteins in the lysosome fractions from HEK293 cells treated with either C9orf72 or control shRNAs under amino acid starvation or stimulation conditions as shown in Fig 4G. RagC levels were normalized against LAMP1 levels. [*p*<0.5, and n = 3 for all groups]. Distribution of data points is presented with mean ± SD.
(TIF)

S6 Fig The schematic diagram for the role of C9orf72 in the regulation of Rag GTPases and TFEB signaling. When normal cells are stimulated with amino acids, Rag GTPases recruit mTOR and TFEB on the lysosome surface where mTOR phosphorylates TFEB. The phosphorylated TFEB is inhibited to translocate into the nucleus where it regulates the downstream genes. C9orf72 interacts with the Rag GTPases and mediates the regulation of mTOR and TFEB function. Upon loss of C9orf72, the Rag GTPase level is decreased on the lysosome, leading to impaired mTOR signaling and reduction of TFEB on the lysosome. As a result, TFEB is abnormally translocated into the nucleus and alters the expression of metabolic genes in the absence of C9orf72.
(TIF)

S1 Table List of primers used for qPCR. (DOCX)
