# Supplementary figures and images for: C9orf72/ALFA-1 controls TFEB/HLH-30-dependent metabolism through dynamic regulation of Rag GTPases
Source: PLoS Genet. 2020 Apr 13;16(4):e1008738. doi: 10.1371/journal.pgen.1008738 (PMC7188304; doi:10.1371/journal.pgen.1008738)

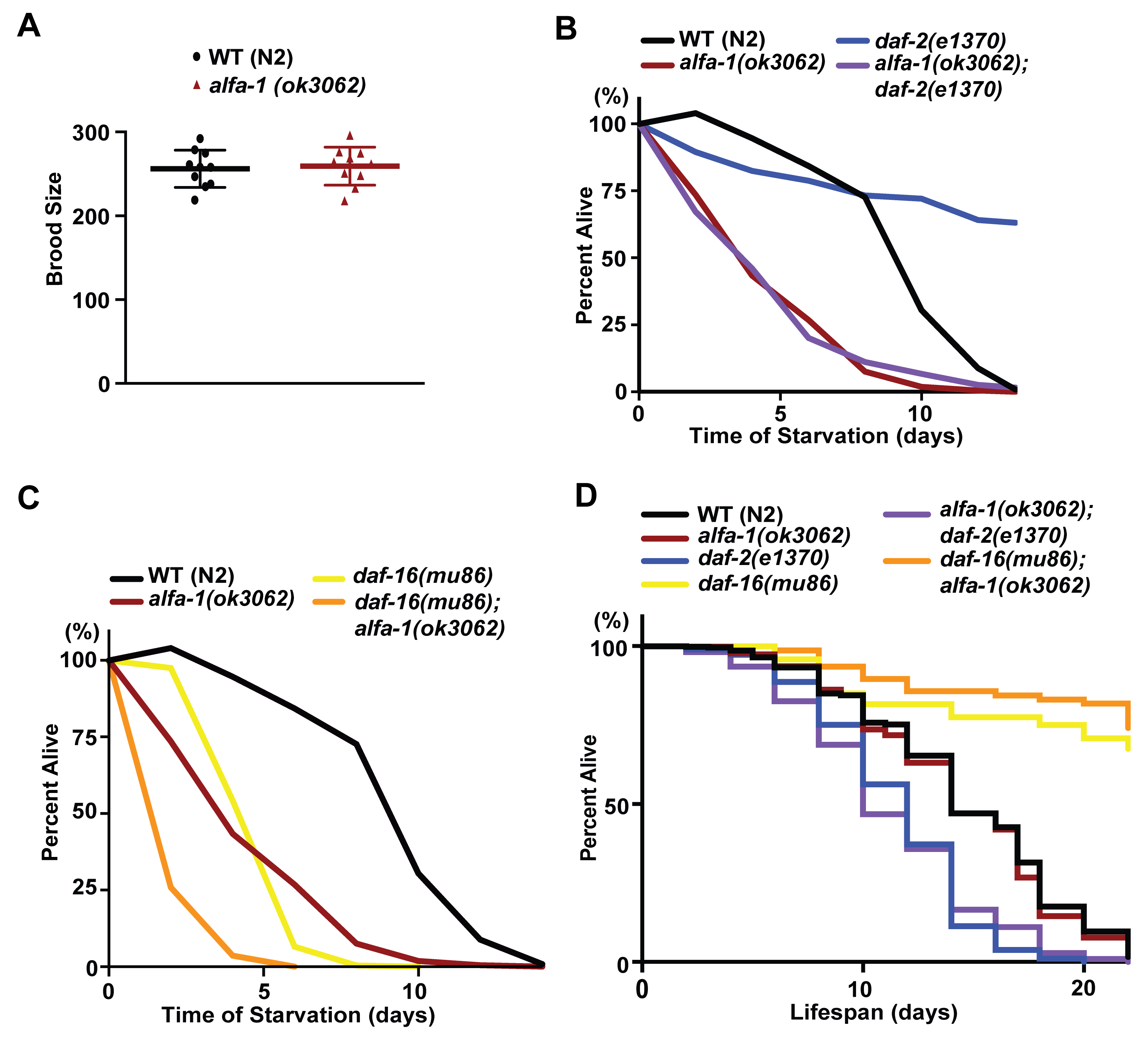

Supplement: S1 Fig — (A) Brood size analysis of alfa-1(ok3062). The total numbers of progeny were counted for the N2 and mutant strains. Distribution of data points are presented with mean ± SD. [p = 0.7679, and n = 10 for all groups) (B) The percentages of wild-type, alfa-1(ok3062), daf-2(e1370), and alfa-1(ok3062);daf-2(e1370) C. elegans surviving to adulthood after incubation of L1 worms in M9 buffer without food. The survival of alfa-1(ok3062) and alfa-1(ok3062);daf-2(e1370) was significantly decreased compared to wild-type worms [p<0.0001], while the survival of daf-2(e1370) was significantly increased [p<0.0001]. The survival of alfa-1(ok3062);daf-2(e1370) was similar to that of alfa-1(ok3062). [p = 0.3305, n = 4502 for N2, n = 4151 for alfa-1(ok3062), n = 2523 for daf-2(e1370), and n = 3257 for alfa-1(ok3062);daf-2(e1370)]. Log-rank (Mantel-Cox) test was used and data are presented as means. (C) The percentages of wild-type, alfa-1(ok3062), daf-16(mu86), and daf-16(mu86);alfa-1(ok3062) C. elegans surviving to adulthood after incubation of L1 worms in M9 buffer without food. The survival of daf-16(mu86) and daf-16(mu86);alfa-1(ok3062) was significantly decreased compared to wild-type worms [p<0.0001], and the survival of daf-16(mu86);alfa-1(ok3062) was significantly decreased compared to alfa-1(ok3062). [p<0.0001, n = 1140 for daf-16(mu86) and n = 352 for daf-16(mu86);alfa-1(ok3062)]. Log-rank (Mantel-Cox) test was used and data are presented as means. (D) Lifespan analysis of alfa-1(ok3062) with daf-2(e1370) or daf-16(mu86) under normal conditions. The lifespan of alfa-1(ok3062) was not changed compared to N2 [p = 0.3428]. The lifespan of daf-2(e1370) was significantly increased, while the lifespan of daf-16(mu86) was significantly decreased compared to N2 [p<0.0001]. The lifespan of alfa-1(ok3062);daf-2(e1370) and daf-16(mu86);alfa-1(ok3062) was similar to that of daf-2(e1370) [p = 0.3675] and daf-16(mu86) [p = 0.7289], respectively. [n = 508 for N2, n = 501 for alfa-1(ok3062), [file pgen.1008738.s001.tif]

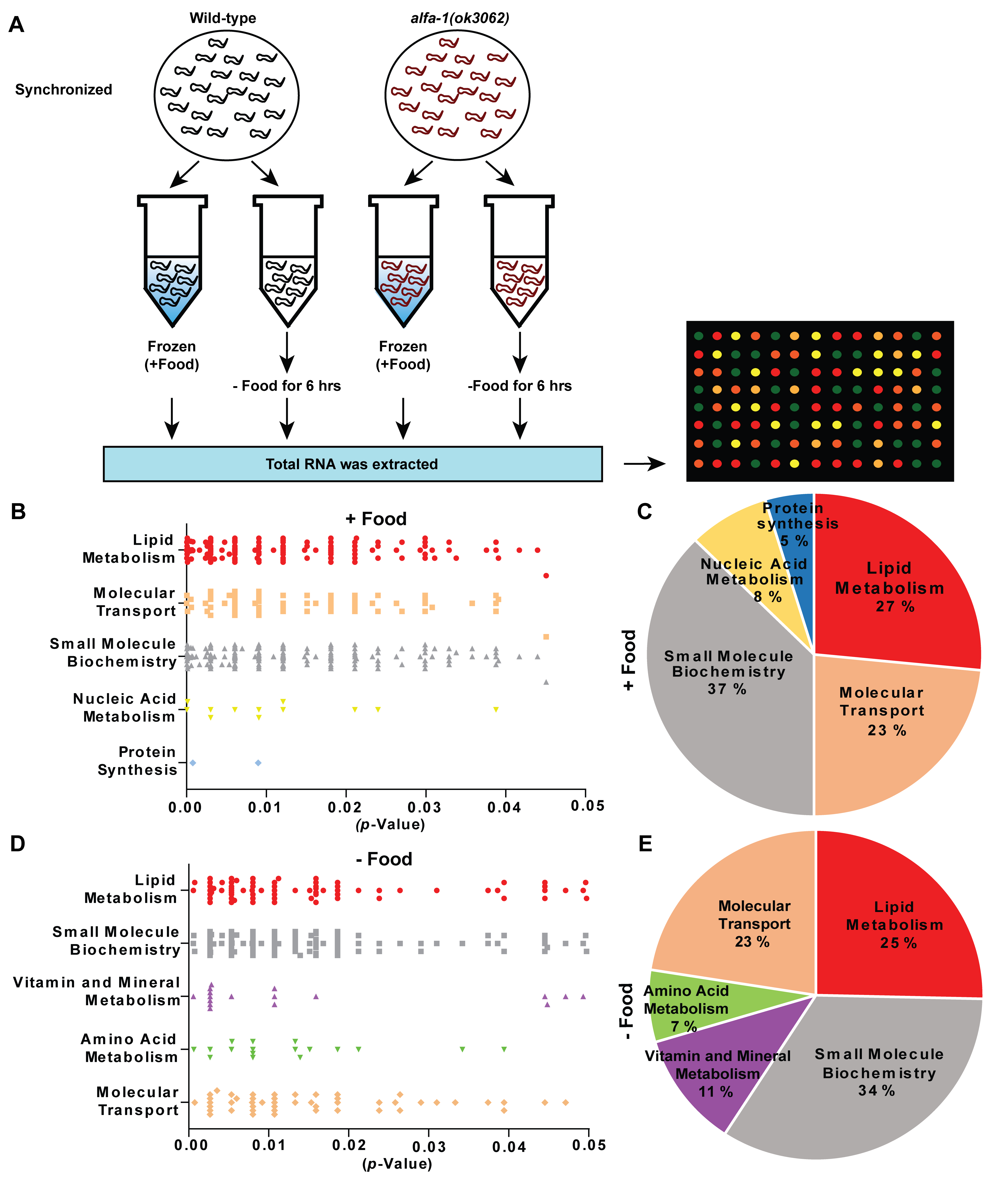

Supplement: S2 Fig — (A) The schematic diagram for the transcriptome analysis of wild-type and alfa-1(ok3062) C. elegans. N2 and alfa-1(ok3062) were synchronized by bleaching and cultured until they reach to L3/L4 stage in the well-fed condition, then the worms equally divided into two groups. One group (+ Food) was frozen for further analysis and the other group (- Food) was starved for 6 hr. Then, the total RNAs were extracted and subjected to the transcriptome profiling using microarrays. (B) The top five molecular and cellular functions of differentially expressed genes in well-fed wild-type and alfa-1(ok3062) mutants. Rankings are given from top to bottom. Each dot represents a gene with a p-value. (C) Percentages of molecules found in (b) related to each molecular and cellular function. (D) The top five molecular and cellular functions of differentially expressed genes in fasted wild-type and alfa-1(ok3062) C. elegans. Rankings are given from top to bottom. Each dot represents a gene with a p-value. (E) Percentages of the molecules found in (d) related to each molecular and cellular function. (TIF) [file pgen.1008738.s002.tif]

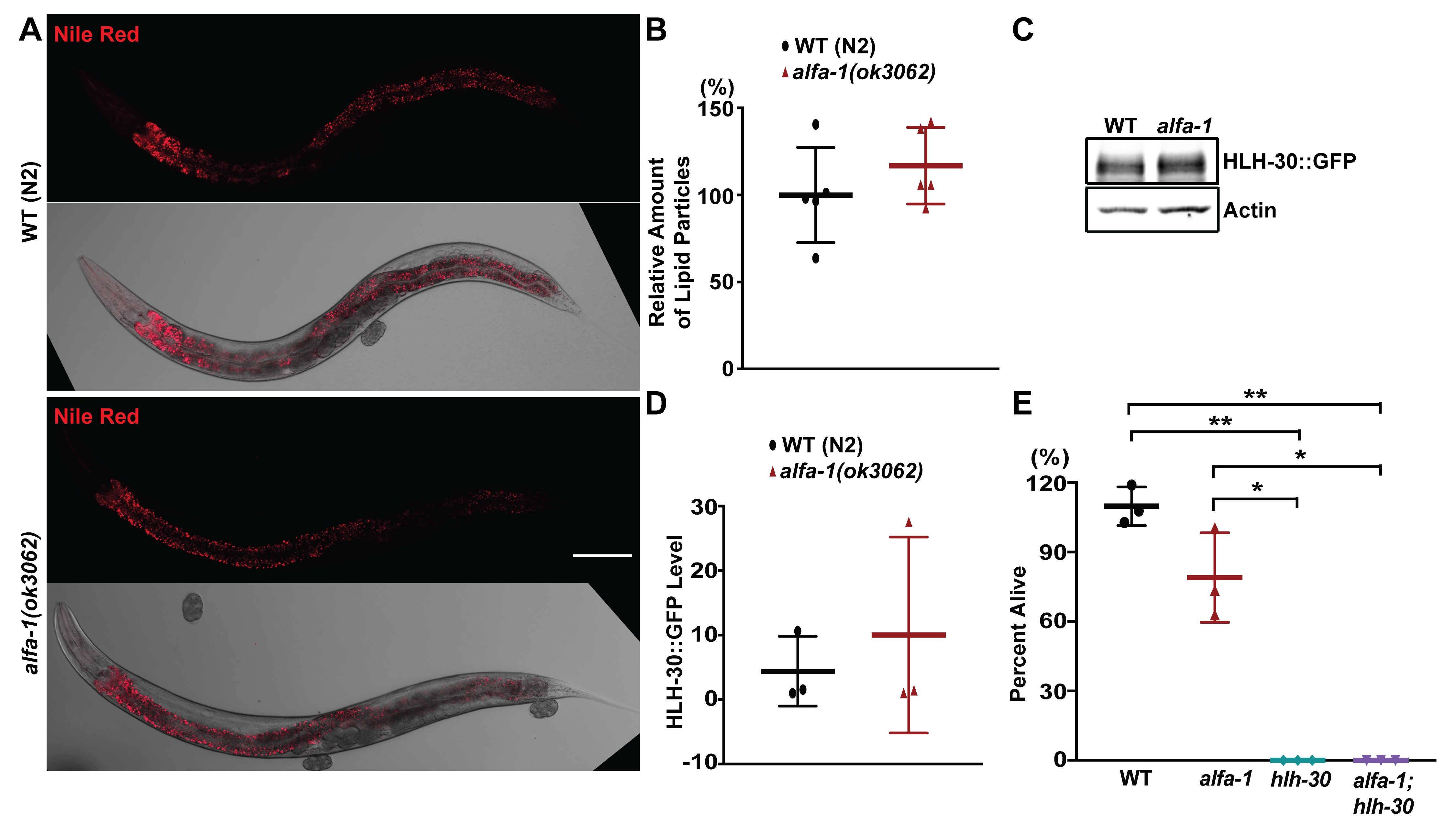

Supplement: S3 Fig — (A) Representative image of Nile Red staining patterns in well-fed adult worms. Both Nile Red staining patterns (upper panels) and DIC images (lower panels) are shown. Scale bar: 100 μm. (B) Quantification of the lipid particles stained with Nile Red in adult wild-type and alfa-1(ok3062) C. elegans under well-fed conditions. [p = 0.3140, n = 5 for all groups]. (C) Immunoblotting of HLH-30::GFP in wild-type and alfa-1(ok3062) worms under starvation conditions. (D) Quantification of HLH-30::GFP levels in (C) with actin as the control. [p = 0.5788, n = 3 for all groups]. (I) The percentages of worms surviving to adulthood after incubation of L1 worms in M9 buffer without food for 2 days for N2, alfa-1(ok3062), hlh-30(tm1978), and alfa-1(ok3062);hlh-30(tm1978) [*p<0.05, and n = 3 for all groups]. Distribution of data points is presented with mean ± SD. (TIF) [file pgen.1008738.s003.tif]

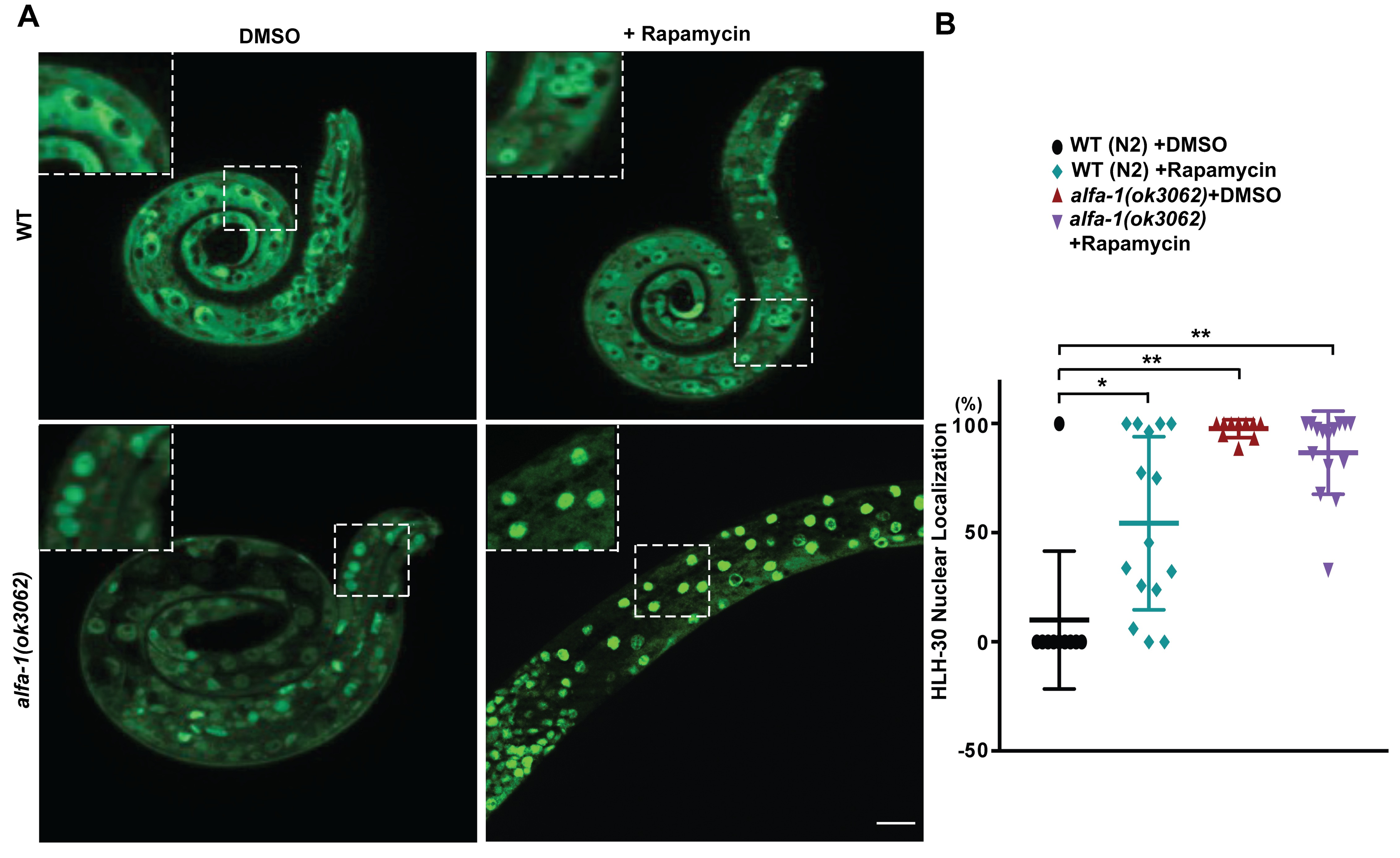

Supplement: S4 Fig — (A) Representative images of HLH-30::GFP in wild-type and alfa-1(ok3062) L1 C. elegans that were treated with DMSO or rapamycin in the absence of food. Enlarged images of boxed areas are shown in each panel. (B) Percentages of nuclear HLH-30::GFP in starved L1 with or without rapamycin. [*p<0.05, **p<0.0001, n = 10 for DMSO groups and n = 15 for rapamycin groups]. Distribution of data points is presented with mean ± SD. Scale bar: 20 μm. (TIF) [file pgen.1008738.s004.tif]

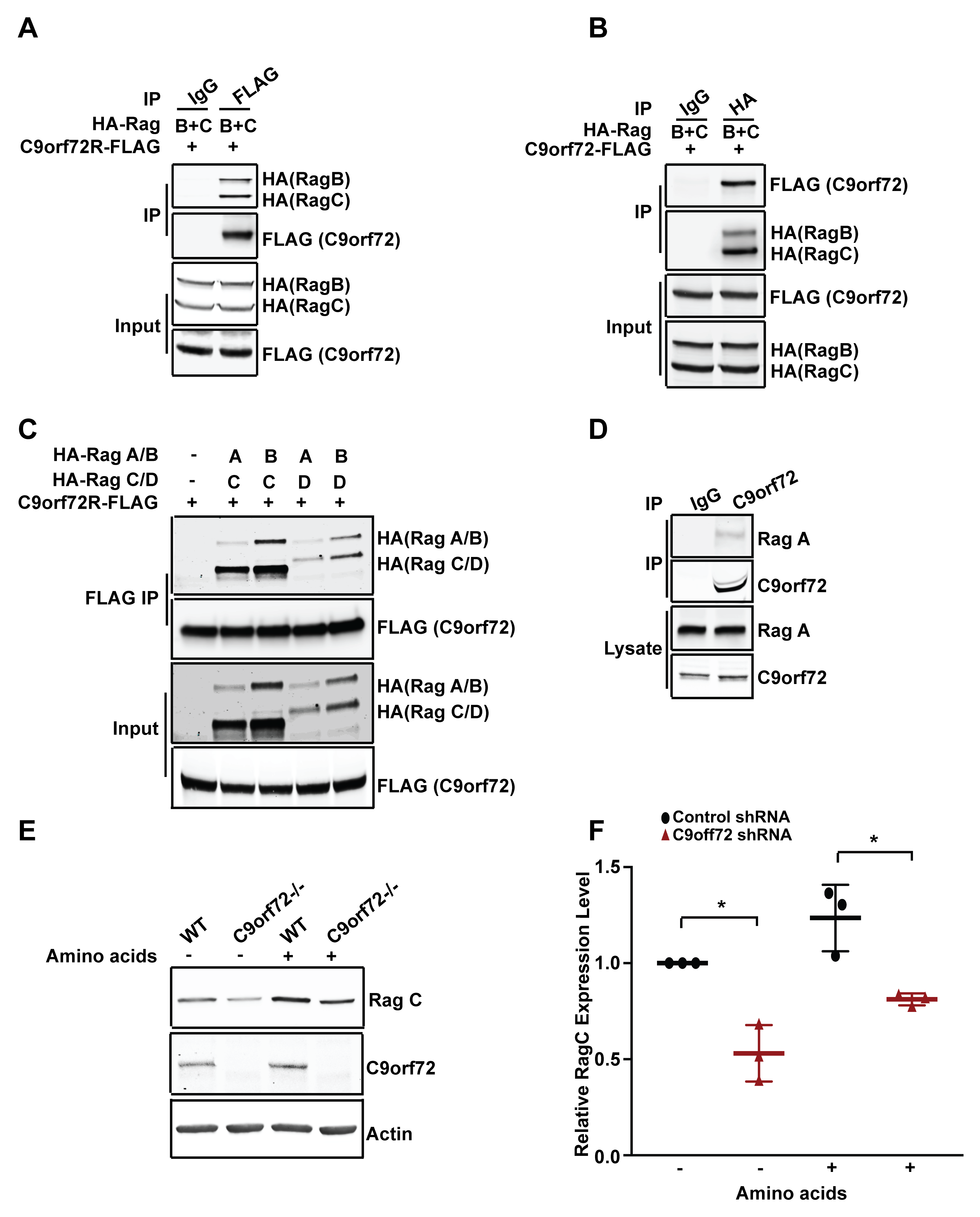

Supplement: S5 Fig — (A) Co-immunoprecipitation analysis of C9orf72-Flag and HA-Rag. HEK293 cells were transfected with the indicated plasmids, cell lysates were prepared at 48 hr post-transfection, and IgG or FLAG-tagged immunoprecipitates were analyzed by immunoblotting with anti-FLAG and anti-HA antibodies. (B) Reciprocal co-immunoprecipitation analysis of HA-Rag and C9orf72-Flag. Cell lysates were prepared as described above, and IgG or HA-tagged immunoprecipitates were analyzed by immunoblotting with anti-FLAG and anti-HA antibodies. (C) Co-immunoprecipitation analysis of C9orf72-Flag and different HA-Rag isoforms. HEK293 cells were transfected with the indicated plasmids, cell lysates were prepared as described above, and IgG or FLAG-tagged immunoprecipitates were analyzed by immunoblotting with anti-FLAG and anti-HA antibodies. (D) Co-immunoprecipitation analysis of C9orf72 and RagA indicates that RagA was detected in C9orf72 immunoprecipitates from HEK293 cells. (E) Immunoblotting analysis showed that RagC protein levels were decreased in C9orf72-/- MEF cells under amino acid starvation or stimulation conditions. (F) Quantification of RagC poteins in the lysosome fractions from HEK293 cells treated with either C9orf72 or control shRNAs under amino acid starvation or stimulation conditions as shown in Fig 4G. RagC levels were normalized against LAMP1 levels. [p<0.5, and n = 3 for all groups]. Distribution of data points is presented with mean ± SD. (TIF) [file pgen.1008738.s005.tif]

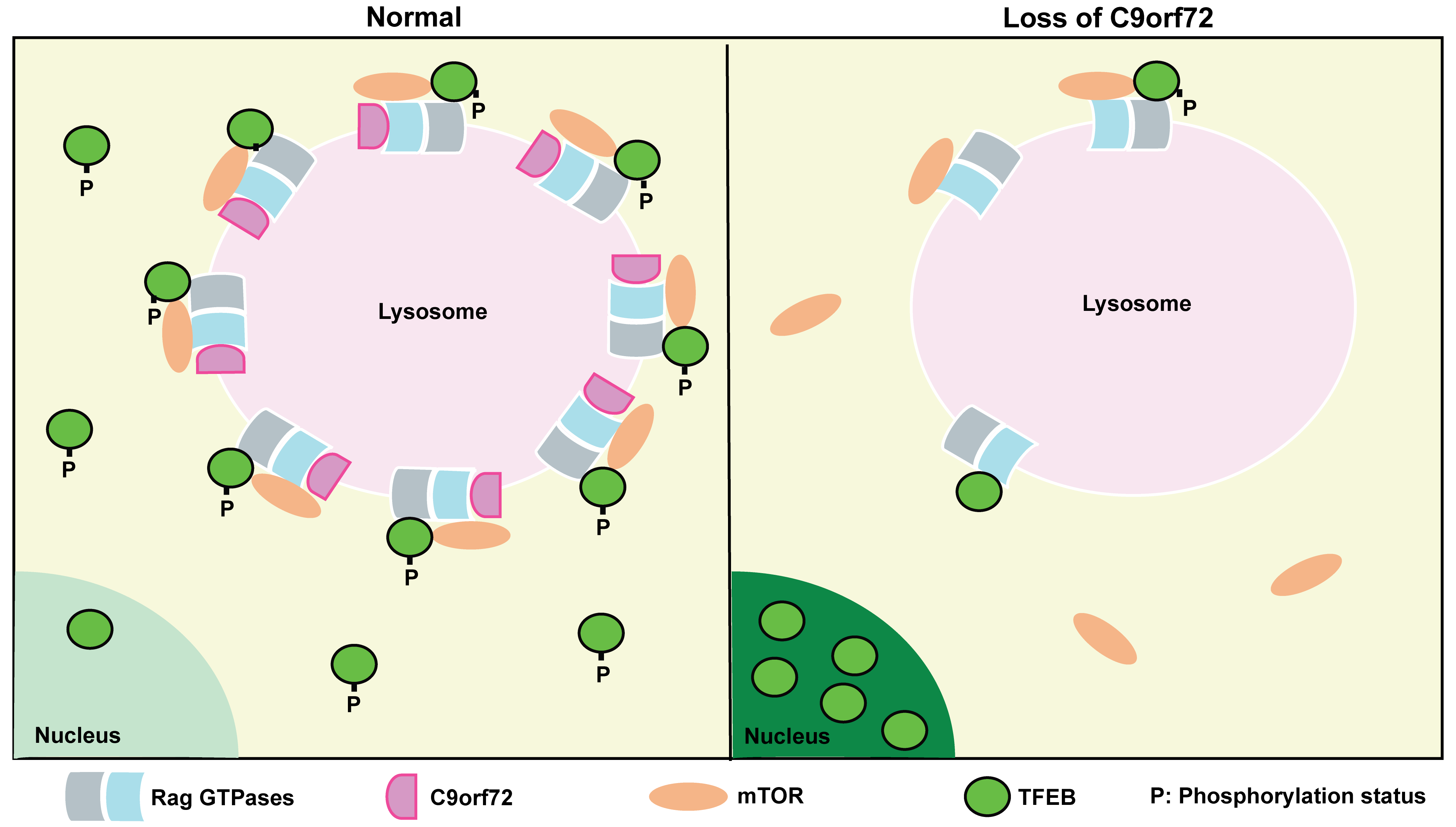

Supplement: S6 Fig — When normal cells are stimulated with amino acids, Rag GTPases recruit mTOR and TFEB on the lysosome surface where mTOR phosphorylates TFEB. The phosphorylated TFEB is inhibited to translocate into the nucleus where it regulates the downstream genes. C9orf72 interacts with the Rag GTPases and mediates the regulation of mTOR and TFEB function. Upon loss of C9orf72, the Rag GTPase level is decreased on the lysosome, leading to impaired mTOR signaling and reduction of TFEB on the lysosome. As a result, TFEB is abnormally translocated into the nucleus and alters the expression of metabolic genes in the absence of C9orf72. (TIF) [file pgen.1008738.s006.tif]
