# Supplementary material for: C9orf72/ALFA-1 controls TFEB/HLH-30-dependent metabolism through dynamic regulation of Rag GTPases
Source: PLoS Genet. 2020 Apr 13;16(4):e1008738. doi: 10.1371/journal.pgen.1008738 (PMC7188304; doi:10.1371/journal.pgen.1008738)
Supplement: S1 Table — (DOCX) [file pgen.1008738.s007.docx]

**Supplemental Table 1. List of primers used for qPCR.**

| GENE | PRIMER SEQUENCES | REFERENCES |
| --- | --- | --- |
| *atg-9* | GGCCGCCATCCACTCATCGG  / TTGACGTCGTGCCGCCGTAG | Lapierre *et al*., 2013^†^ |
| *sqst-1* | TGGCTGCTGCATCATCCGCT  / TCAATCGTGCCGAGACCGGG | Lapierre *et al*., 2013^†^ |
| *lipl-1* | CGGTTTGCGCTGGACTTA  /GAACACGAGTTGCGTTAA | Arvidsson *et al*., 2008^‡^ |
| *lipl-2* | AATACGAGTCAAATCATTGAA  /GTAACACTCGTTTTTCCATAA | Arvidsson *et al*., 2008^‡^ |
| *lipl-3* | ATGGGCAGGCAAATCCACCA  /AGTTGTTCTGCGCAATTATA | Arvidsson *et al*., 2008^‡^ |
| *lipl-5* | CATATGACTACCCCCCAAATCA  /CCATGTTACGTTTGTTTTCCCAAA | Arvidsson *et al*., 2008^‡^ |

^†^ Lapierre LR, De Magalhaes Filho CD, McQuary PR, Chu CC, Visvikis O, Chang JT, et al. The TFEB orthologue HLH-30 regulates autophagy and modulates longevity in Caenorhabditis elegans. Nature communications. 2013;4:2267. doi: 10.1038/ncomms3267. PubMed PMID: 23925298; PubMed Central PMCID: PMCPMC3866206.

^‡^ Samuel Arvidsson, Miroslaw Kwasniewski, Diego M. Riano-Pachon and Bernd Mueller-Roeber: QuantPrime - a flexible tool for reliable high-throughput primer design for quantitative PCR. *BMC Bioinformatics* 2008, 9:465
